# Silver Nanoparticles and Neurotoxicity: Mechanistic Insights and Recent Experimental Evidence

**DOI:** 10.3390/pharmaceutics18050545

**Published:** 2026-04-29

**Authors:** Melis Kaya, Emir Akdaşçi, Furkan Eker, Mikhael Bechelany, Sercan Karav

**Affiliations:** 1 Department of Molecular Biology and Genetics, Çanakkale Onsekiz Mart University, Çanakkale 17100, Türkiye; mlskaya007@gmail.com (M.K.); emirakdasci@stu.comu.edu.tr (E.A.); furkan.eker@stu.comu.edu.tr (F.E.); 2 European Institute for Membranes (IEM), UMR-5635, University Montpellier, ENSCM, CNRS, Place Eugène Bataillon, CEDEX 5, F-34095 Montpellier, France

**Keywords:** silver nanoparticles, neurotoxicity, blood–brain barrier, oxidative stress, mitochondrial dysfunction, neuroinflammation, glial activation, apoptosis

## Abstract

Silver nanoparticles (AgNPs) have gained significant interest across various areas arising from their multifunctional mechanisms. Biomedical applications are one of the areas where the therapeutic and diagnostic potential of AgNPs are highlighted. Considering the expansion of biomedical use of AgNPs, nervous system-based applications, including neuroimaging, neural implant coatings and development of neural tissue-targeted drug delivery systems are some of the potential applications of AgNPs in the current research. However, growing interest in these nervous system related applications and the limited regenerative capacity of neural tissues make it essential to carefully evaluate the potential neurotoxic effects of AgNPs. AgNP-induced responses in neural tissues may differ according to key physicochemical and exposure-related parameters, specifically particle size, shape, surface chemistry, coating properties, protein corona formation, exposure route, dose, and duration. Among the possible mechanisms that may contribute to these responses are blood–brain barrier (BBB) disruption, mitochondrial dysfunction and oxidative stress, neuroinflammation and glial activation, and cell death processes such as apoptosis, autophagy, and ferroptosis. In this review, in the context of the potential neurotoxic effects of AgNPs on the nervous system, the main parameters that determine AgNP neurotoxicity and the possible mechanisms involved are examined in detail, where recent scientific developments in this field are evaluated based on current in vitro and in vivo studies.

## 1. Introduction

Nanotechnology is one of the important and rapidly developing fields of modern science. This technology is leading innovations in numerous fields such as industry, agriculture, food, energy, environment, and health [1,2]. Among these innovations, nanoparticles (NPs) are one of the most studied nanomaterials, exhibiting sizes between 1 and 100 nm [3]. NPs are classified into three main classes according to their origins: organic structures, inorganic structures, and carbon-based structures. Each class has the potential to be tailored for different clinical and biomedical requirements [4,5]. Many organic and polymer-based NPs have been extensively studied for diverse biomedical applications [6,7,8]. However, inorganic NPs stand out among these classes because of their chemical stability, diversity of synthesis methods, and multifaceted physicochemical properties [9]. Inorganic NPs primarily consist of metal nanoparticles (silver [Ag], gold [Au], copper [Cu]) and metal oxide nanoparticles (iron oxide [Fe_3_O_4_], zinc oxide [ZnO], titanium dioxide [TiO_2_], cerium oxide [CeO_2_]) [10,11]. Within this broad class of inorganic NPs, silver nanoparticles (AgNPs) have increasingly become a central focus of interest in recent years [12,13].

AgNPs can be produced using different synthesis methods, including physical, chemical and green synthesis [14,15]. The chosen synthesis method determines the physicochemical parameters of AgNPs, such as their size, shape, surface properties and stability, thereby shaping their overall physicochemical profile. Variations in these parameters can influence the interaction of AgNPs with biological systems, the bioavailability of AgNPs, and the magnitude of the biological response that arises in target tissues [16]. AgNPs can exhibit a range of biological activities, primarily broad-spectrum antimicrobial activity, as well as anticancer, anti-inflammatory, antioxidant, and other biological activities [17]. These versatile biological activities, together with the increased surface-area-to-volume ratios and their suitability for surface functionalization, confer a strong interaction potential in biological environments, making AgNPs a focal point of multidisciplinary research. In the current literature, numerous studies comprehensively examine the use of AgNPs in specific areas such as agriculture, industry, drug delivery systems, wound healing, imaging and various therapeutic applications [18,19,20,21,22].

In parallel with these broad and increasingly diverse application areas, interest in the use of AgNPs in biomedical approaches related to the nervous system has increased markedly in recent years. Numerous studies have suggested that AgNPs can serve as carriers or functional components in neuroimaging, neural tissue-targeted drug delivery systems, neural implant coatings, and in innovative strategies for the treatment of various neurological diseases [23,24,25,26,27,28]. However, the expansion of potential application areas toward critical organs with limited regenerative capacity, such as the nervous system, and the diversification of exposure scenarios significantly alter the risk profile of AgNPs. Thus, the assessment of potential neurotoxic effects of AgNPs is considered extremely important along with the evaluation of their therapeutic potential.

The nervous system, with its central nervous system (CNS) and peripheral nervous system components, forms the fundamental control and integration network of the organism. Structural or functional disturbances within this network can lead to a wide range of neurological symptoms and clinical outcomes [29,30]. In this context, current evidence on AgNP exposure indicates that their effects on the nervous system are not uniform and may vary significantly depending on several parameters. These parameters include physicochemical properties such as AgNP size and shape, surface chemistry and protein corona formation in biological environments. They also include exposure conditions such as the route, dose and duration of exposure [31]. At the mechanistic level, these parameters can shape AgNP interactions with the blood–brain barrier (BBB) and other neural barriers, their uptake by neuronal and glial cells and the resulting intracellular responses. In turn, these effects may promote neurotoxicity-related processes such as disruption of barrier integrity, oxidative stress, neuroinflammation, and various cell death pathways [32,33,34,35]. Therefore, an accurate assessment of AgNP-induced neurotoxic risk requires an integrated evaluation of AgNP effects on the nervous system in light of both these determining parameters and the underlying mechanistic pathways.

In this review, the potential neurotoxic effects of AgNPs on the nervous system are evaluated in an integrated manner based on the current literature. Within this context, the main physicochemical and exposure-related parameters that shape the neurotoxicity profile of AgNPs, such as particle size and shape, surface chemistry, protein corona formation, and exposure route and dose, are first discussed. Mechanisms associated with AgNP exposure, BBB interaction and disruption, mitochondrial dysfunction and oxidative stress, neuroinflammation and glial activation, and cell death pathways such as apoptosis, autophagy and ferroptosis are presented in detail. Subsequently, recent in vitro and in vivo studies have also been reviewed to provide a broader perspective on the neurotoxic potential of AgNPs. Accordingly, this review aims to present both an assessment of the current scientific evidence and a comprehensive framework to inform future applications.

## 2. AgNPs and Neurotoxicity

**Figure 1:**
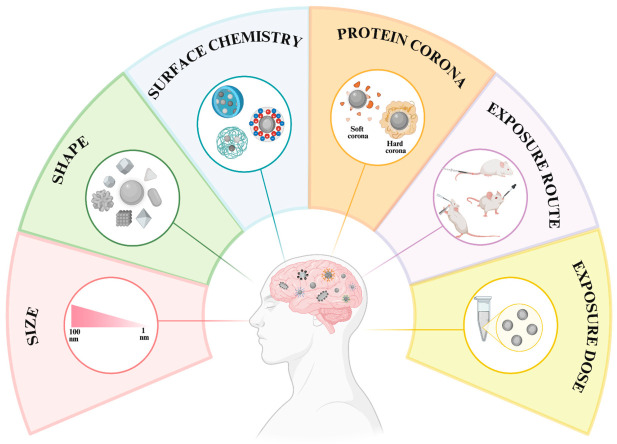
Schematic illustration of the key parameters influencing the neurotoxic potential of silver nanoparticles (AgNPs): physicochemical properties such as size, shape and surface chemistry, protein corona formation, and exposure parameters including route and dose [38,39]. Created in BioRender. Karav, S. (2026) https://BioRender.com/8g4lua3.

Functional or structural impairments of the nervous system, which is referred to as neurotoxicity, can negatively influence both CNS and the peripheral nervous system. Moreover, the limited regenerative capacity and high metabolic demands of the CNS increase its vulnerability [36]. AgNPs’ utilization in the applications involving the nervous system, either directly or indirectly, has been notably increased. Considering the vulnerable nature of the CNS, AgNP-based neurotoxic risks must be carefully evaluated for broader and accurate implementation. The physicochemical properties of AgNPs, such as size, shape, and surface chemistry, can greatly affect their neurotoxic potential. This response may also be influenced by biological identity changes associated with protein corona formation and exposure-related parameters such as route, dose, and exposure time (Figure 1). AgNPs’ ability to cross biological barriers and their interactions with neuronal cells are shaped by these parameters, which eventually promote mechanisms associated with neurotoxicity [37]. Accordingly, a review of these general parameters of toxicity is essential to acknowledge effects of AgNPs on the nervous system.

### 2.1. Size and Shape

Among physicochemical properties particle size has a substantial impact on the neurotoxic potential of AgNPs. Smaller AgNPs exhibit a higher surface-area-to-volume ratio, potentially increasing Ag^+^ ion release and promoting closer contact with cells. Particle size can influence AgNPs’ access to the brain and their distribution within it. Likelihood of AgNPs crossing the BBB is largely inversely proportional to their size. The physical structure of the barrier can substantially restrict the passage of larger particles. Conversely, smaller particles are more likely to cross this barrier efficiently. Thus, as size decreases AgNP brain penetration efficiency and access to vulnerable brain regions may increase. Once inside the brain, smaller particles may exhibit broader distribution and higher accumulation. Such conditions can markedly increase overall neurotoxic risk. Additionally, smaller particles can lead to higher surface reactivity. This may trigger multiple cellular mechanisms associated with neurotoxicity [38].

For instance, Patchin et al. examined how AgNP neurotoxicity varies with particle size [40]. In this study, rats were exposed to citrate-coated AgNPs of 20 nm and 110 nm via inhalation. The distribution and persistence of AgNPs were monitored in the nasal epithelium and olfactory bulb for up to 56 days. Microglial activation was also measured as an indicator of neuroinflammation. As a result smaller AgNPs dissolved more rapidly, accumulated more extensively in the olfactory bulb and promoted stronger microglial responses compared to larger particles. Although this study focuses on the olfactory route, it supports the principle of size-dependent barrier crossing discussed above. The ability of smaller NPs to cross physical barriers more easily may contribute to increased tissue accumulation. Accordingly, observed neurotoxic responses, including microglial activation, may become more pronounced.

In addition to particle size, particle shape is another physicochemical factor that may influence the neurotoxic potential of AgNPs. Depending on synthesis conditions, AgNPs may be produced in spherical, cubic, triangular, rod-like, and wire-like forms [41]. Particle shape can alter protein corona composition and how AgNPs interact with the cell membrane through differences in surface facets and edge/corner density, which can affect cellular uptake. Although neuro-specific data are limited, available studies suggest that shape may influence cellular mechanisms underlying neurotoxicity [37].

For example, a recent study by Kumarasamy et al. demonstrated an association between AgNP shape and neurotoxicity [42]. In this in vitro study, PVP-coated spherical, cubic, rod-shaped and triangular AgNPs with a size of approximately 50 nm were tested on NE-4C neural stem cells. The analyses revealed that rod-shaped AgNPs reduced cell viability below 20% even at 1 µg/mL. In contrast, cubic AgNPs exhibited similar toxicity only at 50 µg/mL. Triangular AgNPs were observed to exhibit mild toxicity of less than 50% at 100 µg/mL. Spherical AgNPs showed no marked effect. The higher toxicity of these rod-shaped and cubic AgNPs is attributed to their greater protein adsorption capacity and increased Ag^+^ ion release at edge and corner regions. These findings highlight the importance of particle shape in determining the neurotoxicity of AgNPs.

Considering the effect of synthesis conditions, such as pH, temperature and AgNO_3_ concentration, on the physicochemical properties of AgNPs, their influence on the neurotoxic potential should not be overlooked [43].

### 2.2. Surface Chemistry

Coating agents, functional groups, surface charge and hydrophobicity are the primary determinants of AgNPs’ surface characteristics. For instance, while the coating agents affect oxidation and ion release through alteration of the colloidal stability, surface charge can regulate the electrostatic interactions with cell membranes and cellular uptake processes. Furthermore, positively charged surfaces generally interact strongly with negatively charged cell membranes that promote cellular uptake and more sensitive toxic responses. Negatively charged surfaces, on the other hand, interact less with cells and tend to be less toxic [44,45]. The context dependent surface chemistry can lead to variations in toxicity across different environmental conditions, cell types, and coating materials.

Surface chemistry can also influence AgNPs’ stability, agglomeration propensity, ion-release kinetics and behavior in biological environments in addition to cell level interactions [46]. These biophysical changes can lead to AgNPs triggering cellular stress responses. Due to its high energy requirements and limited regenerative capacity, these responses may cause more pronounced effects in brain tissue [47]. Therefore, differences in surface chemistry are suggested to shape the biological interactions of AgNPs and associated neurotoxic processes.

To clarify the role of surface charge in AgNP-cell interactions, Barbalinardo et al. investigated how charge polarity impacts cellular uptake, intracellular distribution, and toxicity [48]. For this purpose, murine fibroblasts (NIH-3T3) and human cancer cells (MCF7 and Caco2) were exposed to AgNPs coated with negatively charged citrate (−42 ± 2 mV) and PSS (−56 ± 2 mV) or positively charged PAH (+36 ± 4 mV) and PDDA (+44 ± 4 mV). Analyses revealed that positively charged AgNPs exhibited higher cellular uptake than negatively charged AgNPs in all three cell lines. This difference was ascribed to stronger electrostatic interactions with negatively charged cell membranes. Positively charged AgNPs, accumulating particularly within lysosomes, triggered severe cytotoxic effects leading to necrosis through pronounced mitochondrial damage. Conversely, negatively charged AgNPs exhibited minimal cellular uptake inducing only minor mitochondrial disruptions and early apoptotic events. Overall, these findings indicate that coating-derived surface charge can influence AgNP toxicity by modulating membrane interactions, cellular uptake and intracellular distribution. Although this study was not conducted in neural cells, these charge dependent cell–particle interactions may be highly relevant for interpreting neurotoxic responses. This is because neuronal cells are highly susceptible to mitochondrial dysfunction and the disruption of intracellular homeostasis. Furthermore, cell–particle interactions may influence AgNP contact with neural barriers and their potential to cross them. Therefore, these findings provide a general mechanistic basis for interpreting coating dependent toxic responses observed in neural models.

Pongrac et al. evaluated the neurotoxic effects of AgNPs with different surface coatings on murine neural stem cells (mNSCs) across a broad spectrum of surface charges [49]. In this study, the cellular uptake and toxicity profiles of AgNPs coated with positively charged PLL (34.5 ± 2.8 mV) and CTAB (42.6 ± 5.1 mV), negatively charged AOT (−32.3 ± 2.6 mV), and the neutral coatings PVP and BSA were compared. The analyses revealed that the most severe cytotoxicity occurred in the positively charged PLL and CTAB groups. At a dose of 5 mg Ag/L, these groups reduced cell viability by more than 85%. By contrast, cell viability for the negatively charged AOT and neutral PVP coatings remained at approximately 50% at the same concentration. This marked increase in toxicity in the positive group is thought to result from the strong electrostatic interaction of positively charged particles with the negatively charged cell membrane. When examined on the basis of specific chemical groups, surfactant-based coatings (CTAB and AOT) were found to exhibit a structurally more destructive tendency toward neural cells compared to their polymer-based counterparts (PLL and PVP). These findings demonstrate that surface charge polarity and specific chemical groups directly influence the severity of toxicity and the interaction mechanisms in neural cells.

For example, Begum et al. examined the toxic effects of AgNPs with different surface coating materials on neuronal cells [50]. For this purpose, human embryonic stem cell-derived glutamatergic neurons were exposed for 72 h to 20 nm AgNPs coated with either citrate (AgSC, −48 mV) or PVP (AgSP, −37 mV) at concentrations from 0.1 to 5 µg/mL. A dose-dependent decrease in cell viability was observed for both types of AgNPs. However, AgSCs with a higher zeta potential were found to accumulate more in neurons and to induce more pronounced neurotoxicity compared with AgSPs. Moreover, an increase in reactive oxygen species and a disturbance in Ca^2+^ homeostasis were evident in both groups. A decrease in neuronal markers (MAP2, PSD95, vGLUT1, and NMDA) and neurite loss were also recorded. However, these changes were more pronounced in the AgSCs group. Overall, these findings indicate that differences in surface coating and charge can influence AgNP-neuron interactions, intracellular accumulation, and associated neurotoxic responses.

Dziendzikowska et al. investigated the effects of AgNPs with different surface coating materials on cognitive functions, memory processes, and neurotransmitter levels [51]. In this study, male Wistar rats were orally administered AgNPs coated with bovine serum albumin (AgNP-BSA), polyethylene glycol (AgNP-PEG), citrate (AgNP-Cit), and AgNO_3_ (Ag^+^ ions). All groups received 0.5 mg/kg b.w. of silver for 28 days. Behavioral tests revealed that spatial learning and long-term memory performance were significantly reduced in the AgNP-BSA, AgNP-PEG, and Ag^+^ groups compared to the control. By contrast, cognitive functions were largely preserved in the AgNP-Cit group. Neurotransmitter analysis revealed alterations in the serotonin-to-dopamine ratio and in acetylcholine levels in the AgNP-BSA group. Taken together, these findings suggest that the surface coating of AgNPs is an important factor influencing their neurotoxicity.

In summary, surface chemistry can greatly affect the neurotoxic potential of AgNPs. Variances in surface chemistry can modulate NP interactions in biological systems and influence cellular responses. Understanding how surface chemistry affects neurotoxicity is important for the safe use of AgNPs.

### 2.3. Protein Corona

Beyond physicochemical properties, another parameter that can affect the neurotoxicity of AgNPs is the protein corona (PC). Proteins, peptides, and other biomolecules rapidly adsorb onto the surface of a nanoparticle when it contacts biological fluids such as plasma, blood, cerebrospinal fluid, or culture medium. Accordingly, a PC layer is formed that influences how the nanoparticle is recognized by biological systems and confers it a new biological identity. The formation of the PC is a dynamic process. Competitive adsorption between the NP surface and proteins in the environment leads to a reshaping of protein distribution over time. During this process, low affinity proteins initially bind to the surface and are later replaced by proteins with higher affinity. This is known as the Vroman effect. Thus, two types of corona can develop on the nanoparticle surface. A hard corona consisting of tightly bound yet partially reversible proteins and a soft corona comprising more loosely bound proteins that exchange rapidly [52]. In complex biological environments such as human blood, the binding sequence of different proteins is important. Human blood contains various proteins including albumin, immunoglobulin, fibrinogen, apolipoprotein, transferrin, complement proteins, and hemoglobin. Among these, albumin and fibrinogen are thought to be the first proteins adsorbed onto the NP surface [53]. Moreover, the composition and structure of the corona formation can be influenced by intrinsic properties such as NP diameter, shape, and surface chemistry, as well as by environmental conditions including exposure time, protein concentration, and pH. Particle size, which affects surface curvature, may influence the conformational adaptation of proteins and their spatial organization on the nanoparticle surface [54].

Spagnoletti et al. evaluated the impact of surface coating and protein corona differences on the stability and toxicity of AgNPs by comparing biologically and chemically synthesized nanoparticles [55]. In experiments using *Caenorhabditis elegans*, exposure to 100 mg/L of chemically synthesized AgNPs almost completely reduced growth, reproduction, and the formation rate of gravid adults. At the same concentration, no marked toxicity was observed in biologically synthesized AgNPs coated with natural proteins, with respect to growth and gravid adult formation. These results show that structural differences in the protein corona can modulate the biological behavior and toxicity of AgNPs.

The presence and composition of the protein corona can influence particle stability, agglomeration behavior, and ion release rates. Such alterations can modulate neurotoxicity-related cellular responses. Moreover, the structure of the protein corona can affect the intensity of these cellular responses. While reducing the direct contact with the AgNP surface can lead to the reduction in toxic effects, enhanced transportation of reactive Ag^+^ ions may increase toxicity [37].

The effects of protein corona formation in serum containing environments to chemical transformations on the surface of AgNPs were investigated [56]. Quantitative estimations revealed that 15 to 40% of the silver in AgNPs was converted into silver sulfide (Ag_2_S) under serum containing conditions. A marked reduction in toxicity was observed for AgNPs coated with Ag_2_S following in vivo assays. This demonstrated that protein corona formation can modulate chemical processes on the surface of AgNPs and reduce their toxicity.

Barbir et al. showed that the protein corona formation on AgNPs can increase toxicity under certain conditions [57]. AgNPs coated with albumin (ALB) and metallothionein (MT) were compared with PVP-coated AgNPs. The results showed that both ALB-coated and MT-coated AgNPs induced a more pronounced oxidative stress response than PVP-AgNPs. This difference was particularly evident in female Wistar rats. In the alkaline comet assay, females exhibited increased tail intensity in blood and liver after exposure to all AgNP types. However, in the kidney a significant increase was found only in the ALB-AgNP group. Overall, under certain conditions, the protein corona can increase toxicity potential through modulation of biodistribution and tissue level responses.

Studies directly investigating how changes in protein corona composition affect the neurotoxicity of AgNPs remain limited compared to those focusing on their general toxicological effects. The current evidence suggests that processes such as corona-dependent changes in stability, bioavailability, and cellular recognition may influence the interaction of AgNPs with neural tissue. However, more comprehensive studies are needed to understand the neurological outcomes of these effects.

### 2.4. Exposure Route and Dose

The neurotoxic potential of AgNPs is also influenced by the exposure route and dose. AgNPs can enter the organism mostly through inhalation, nasal, oral, intravenous and to a limited extent, dermal routes. Each of these routes can affect the transport of particles to the neural tissues, their bioavailability, and the severity of their toxic effects in different ways. Therefore, each exposure route should be assessed separately for its impact on neurotoxicity [58,59].

Among the different routes of exposure, inhalation and intranasal exposure represent the most direct pathways for reaching the brain. These routes can result in the transport of AgNPs along the olfactory nerve, bypassing systemic circulation and directly reaching the olfactory bulb. Huynh et al. investigated how inhalation exposure to aerosolized silver silica nanoparticles (Ag-SiO_2_ NPs) affected microglial activation in the olfactory bulb of rats [60]. In the study, rats were exposed to aerosolized Ag-SiO_2_ at a concentration of 4.9 ± 2.3 mg/m^3^ for 6 h, Microglial activation in the olfactory bulb was significantly elevated on days 1 and 7, returning to control levels by day 21. These findings suggest that inhaled AgNPs may reach the CNS through the olfactory system and induce a temporary neuroinflammatory response. In another example, Liu et al. investigated the accumulation of Ag-containing particles in the rat brain following the intranasal administration of a nasal spray containing AgNPs [61]. Mass concentrations of Ag-containing particles were measured in the olfactory bulb, striatum–midbrain–brainstem (SMS) region, hippocampus and cerebral cortex using spICP-MS. The highest accumulation was detected in the cerebral cortex at week 4. The measured level was 17.26 ng/g. These findings suggest that intranasal exposure may facilitate the direct transfer of silver-containing particles to the brain.

Oral administration is another route of exposure that allows AgNPs to cross the BBB after systemic absorption and accumulate in the brain. In this process, dissolution in the gastrointestinal tract may increase and ions entering the systemic circulation may be transported to the brain. Recordati et al. investigated the effects of repeated oral administration of 10 nm citrate-coated AgNPs on the brains of mice [62]. At the end of 4 weeks, the brain was identified as the organ with the highest concentration of silver accumulation. The level of accumulation in brain tissue is approximately 0.02–0.03 µg/g in the high-dose AgNP (1 mg/kg) group. Along with silver accumulation in brain tissue, signs of glial activation and mild ultrastructural disruption of BBB integrity were observed. These findings suggest that, although oral exposure is an indirect route, it may still facilitate the transfer of AgNPs to the brain and influence potential neurotoxic processes.

Intravenous and dermal/transdermal exposures are among the routes of administration used for the transfer of nanoparticles into the body. Intravenous administration occurs through the direct delivery of nanoparticles into the circulatory system, most commonly via injection. Among all exposure types, this route results in the greatest systemic bioavailability. Dermal exposure is usually limited by the protective function of the skin barrier; however, prolonged contact or impaired skin integrity may allow nanoparticles to enter systemic circulation [63]. Nevertheless, experimental studies that directly reveal the neurotoxic effects of AgNPs via dermal exposure remain limited. This should be considered as an important area that needs to be investigated in future studies. Dan et al. conducted a study on a rat model to investigate the neurotoxic effects of intravenously administered AgNPs [64]. A single dose (5 mg/kg) of AgNP suspension was administered to Sprague-Dawley rats via the intravenous route, and it was evaluated 24 h later. The evaluations showed no significant change in BBB permeability and confirmed the presence of silver in brain tissue. Astrocyte endfoot swelling, neuronal shrinkage, and particles consistent with AgNPs within neurons were observed using TEM analysis. In addition, transcriptome analyses releaved that the MAPK and Ca^2+^ signaling pathways were affected, and that the expression of genes associated with these pathways changed significantly. These findings indicate that AgNPs can reach brain tissue following intravenous exposure and influence inflammatory and apoptotic pathways.

Although the way AgNPs reach the CNS largely depends on the route of exposure, the severity and extent of their neurotoxic effects mainly vary according to the administered dose and the duration of exposure. Long-term exposure at low doses can lead to slow but persistent accumulation of silver in neural tissue; whereas high doses can rapidly trigger pronounced cellular stress and behavioral impairments [65]. Hence, dose-dependent responses should be carefully examined when evaluating the neurotoxic effects of AgNPs.

In one detailed study that illustrated this relationship, Agwa et al. investigated the effects of orally administered starch-coated AgNPs at different doses (10 µg/kg–10 mg/kg/day) over 90 days on the rat brain [66]. A marked decrease in learning and memory performance at doses of 500 µg/kg and above, a reduction in dopamine levels starting from 10 µg/kg, and disturbances in serotonin and acetylcholinesterase activity starting from 100 µg/kg were detected. Also, at higher doses, increases in oxidative stress markers and neuronal degeneration were observed. Even though there was a notable increase in the neurotoxic potential of AgNPs in dose-dependent manner, the neurotoxic effects may occur even at low doses.

Tareq et al. evaluated the effects of the oral administration of green-synthesized AgNPs on the rat brain [67]. AgNPs were administered to rats at varying doses of 0.5, 5 and 10 mg/kg. After 14 days, all AgNP treated groups demonstrated reduction in acetylcholinesterase activity on both cortex and hippocampus. At the highest AgNp concentration, 34% and 30% reduction in serotonin and norepinephrine levels were detected, respectively. Still, oxidative stress markers remained unchanged. These findings indicate that changed synthesis methods of AgNPs can still affect nervous system function at high doses.

The exposure route and dose can influence the efficiency of how AgNPs reach the CNS, as well as their bioavailability and the severity of potential toxic responses. Certain exposure routes allow direct transportation of AgNP to the nervous system, while higher doses and longer exposure durations can increase the severity of the neurochemical and structural impairments. Thus these parameters are important for the assessment of the neurotoxic effects and to determine their safe exposure levels.

In summary, the neurotoxic effects of AgNPs are shaped by their physicochemical properties, protein corona formation, along with exposure conditions such as route, dose, and duration (Figure 1). These parameters can modulate the AgNPs’ ability to cross biological barriers, their interaction with neural cells and accumulation. Through these effects, molecular processes are modulated and this can lead to structural and functional changes in neural tissue. Thus, these parameters should be considered together and optimized to ensure the safe use of AgNPs.

## 3. Mechanistic Pathways of AgNP Neurotoxicity

### 3.1. Blood–Brain Barrier Interaction and Disruption

An important aspect of assessing the neurotoxic potential of AgNPs is studying their mechanistic pathways on the nervous system. For these mechanisms, explaining how AgNPs enter the CNS is the first and critical stage of understanding their neurotoxicity. AgNPs can be influenced by many parameters as also discussed in the previous section (Figure 1). These parameters can facilitate AgNP access to the CNS and thereby contribute to disruption of barrier integrity and neurotoxic effects. NPs can reach the brain through two main pathways. The first is the neural transport pathway. Following inhalation or intranasal exposure NPs can travel along the olfactory and trigeminal nerves and reach brain tissue directly from the nasal cavity. The other is the systemic circulation pathway, through which NPs, after entering the bloodstream, can reach brain tissue by crossing the BBB [36].

The BBB is a dynamic barrier composed of brain endothelial cells interconnected by tight junctions. This barrier regulates the entry of substances in a highly selective manner to maintain CNS homeostasis [68]. The pathways through which NPs cross the BBB are generally classified into three principal mechanisms. The first of these mechanisms is transcytosis, a vesicular transport process that occurs through brain endothelial cells. Transcytosis is generally classified into three sub-pathways: adsorptive transcytosis, receptor-mediated transcytosis, and carrier-mediated transport. Adsorptive transcytosis occurs through the binding of nanoparticles to the endothelial cell membrane via their surface charges and their transport within small vesicles. In receptor-mediated transcytosis, NPs interact with receptors for ligands such as transferrin or insulin via surface-bound ligands and can cross the barrier. Carrier-mediated transport uses endogenous membrane proteins, such as glucose transporters (GLUT1) and amino acid transporters (LAT1), and is particularly important for NPs with surfaces modified with biomolecules. As another mechanism, paracellular (intercellular) transport refers to the passage of NPs into brain tissue through gaps between endothelial cells due to the weakening or disruption of tight junctions. Although paracellular transport is limited under normal conditions, it can markedly facilitate the passage of NPs in conditions such as inflammation or trauma. The final mechanism is cell-mediated transport, also known as the Trojan horse mechanism. During this mechanism, immune cells such as monocytes or macrophages internalize NPs and transport them across the BBB into brain tissue [69,70].

**Figure 2:**
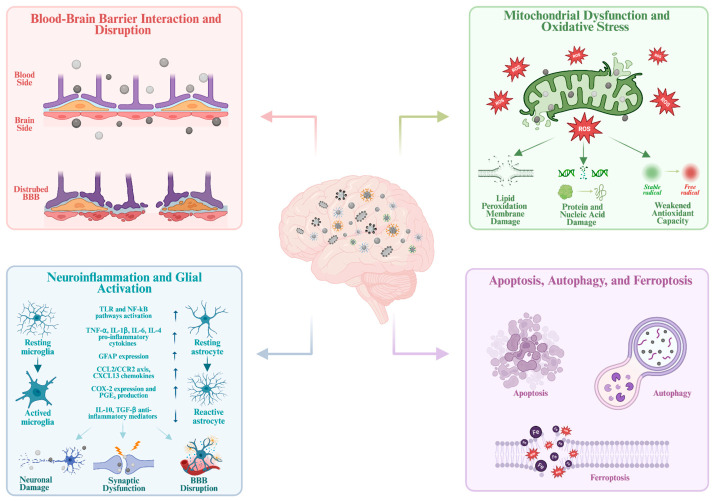
Schematic illustration of the main molecular mechanisms of AgNPs-induced neurotoxic damage, including blood–brain barrier (BBB) interaction and disruption, mitochondrial dysfunction and oxidative stress, neuroinflammation and glial activation, and apoptosis, autophagy, and ferroptosis [47,75]. Created in BioRender. Karav, S. (2026) https://BioRender.com/xtbjyjj.

AgNPs can cross the BBB through these mechanisms and may even disrupt its structural and functional integrity. AgNPs can interact with the endothelial cells that form the BBB and, over time, disrupt the barrier’s physiological balance (Figure 2). The prolonged retention of AgNPs in the brain may contribute to the development of a microenvironment that challenges the barrier’s delicate structure and creates pressure. This microenvironment may provide suitable conditions for the initiation of processes that increase permeability and exacerbate the potential of AgNPs to affect barrier integrity [71].

For example, in the triple co-culture BBB model (endothelial-pericyte-astroglia) developed by Xu et al., the cells were exposed to 10 µg/mL AgNP [72]. After 24 h following the exposure, a significant decrease in transendothelial electrical resistance (TEER) values and a reduction in the expression of the tight junction protein ZO-1 were observed. Additionally, mitochondrial shrinkage, endoplasmic reticulum expansion, and discontinuous tight junction regions were detected in endothelial cells in TEM examinations.

Similarly, in an in vitro study conducted by Tang et al., it was determined that after 4 h of exposure to AgNPs with a size of 70 ± 20 nm at a concentration of 100 µg/mL, approximately 2.76% of the nanoparticles crossed the artificial BBB model, and 34.12% accumulated in endothelial cells [73]. Furthermore, TEM examinations revealed ultrastructural damage, such as relaxation of tight junction gaps, mitochondrial swelling, loss of granules, and endoplasmic reticulum degeneration in some cells.

In the study by Trickler et al., exposure of primary rat brain microvascular endothelial cells to 25 nm AgNPs at 50 µg/mL led to about a 7-fold increase in IL-1β levels and a marked rise in the release of TNF-α and PGE_2_ [74]. This inflammatory response increased the permeability of the endothelial barrier by approximately 1.9 times, leading to a disruption in barrier integrity.

In an in vivo study conducted by Sharma et al., AgNPs were administered intravenously to rats at a dose of 30 mg/kg [75]. Measurements at 24 h showed increased Evans blue extravasation and brain edema. Overall, these studies demonstrate that AgNPs disrupt the structure of the BBB and increase its permeability. Such barrier disruption may facilitate particle entry into the neural environment and subsequently trigger intracellular toxicity.

### 3.2. Mitochondrial Dysfunction and Oxidative Stress

Along with the disruption of BBB integrity, AgNPs reach brain tissue across the BBB and set the stage for the emergence of intracellular effects that initiate the neurotoxicity process. At this stage, AgNPs can accumulate in neuronal cells, causing marked disruptions in cellular energy metabolism and oxidative balance. In particular, the impairment of mitochondrial functions and the release of silver ions (Ag^+^) play an important role in the oxidative stress induced by AgNPs. This oxidative state is further exacerbated by the excessive generation of reactive oxygen species (ROS) [76].

AgNPs can lead to disruption of mitochondrial membrane integrity, a reduction in transmembrane potential and electron transport chain dysfunction. These processes disrupt cellular energy metabolism and contribute to increased oxidative stress. In addition, Ag^+^ released from lysosomes after endocytosis can increase the intracellular oxidative burden. This increase may contribute to the mitochondrial dysfunction, which can disrupt intracellular calcium homeostasis through affecting Ca^2+^ storage and buffering capacity. Excessive intracellular calcium accumulation accelerates ROS generation, further damaging the mitochondrial membrane. As a result, release of cytochrome c and other proapoptotic proteins are promoted, activating cell death pathways with apoptosis or necrosis [76]. High energy requirements and limited regenerative capacity of neuronal mitochondria cause them to be particularly sensitive to environmental influences. Mitochondrial dysfunction is therefore considered a major neurotoxic mechanism of NP exposure [77].

Effects of the exposure to low dose AgNPs on early mitochondrial changes were investigated by Skalska et al. on the adult rat brains [78]. In this study, 10 nm citrate stabilized AgNPs were administered orally at a dose of 0.2 mg/kg for 14 days. Ultrastructural analysis revealed marked swelling of neuronal mitochondria and loss of cristae integrity. Additionally, nanosized granules compatible with AgNPs were detected within the mitochondrial matrix, highlighting the direct localization of particles within the membrane. Consistent with these structural changes, mitochondrial membrane potential decreased by approximately 29% compared to the control group. In addition, changes in the mitochondrial morphology and characteristics associated with early autophagy were observed.

Oxidative stress resulting from AgNP-induced mitochondrial dysfunction plays a key role in neuronal damage, as increased ROS generation and Ag^+^ ion release can impair the glutathione-based antioxidant defense system. This disruption can accelerate lipid peroxidation and promote accumulation of products such as malondialdehyde (MDA). It can also damage cell membrane integrity [46]. ROS causes oxidative damage to proteins and nucleic acids, which accumulates due to limited antioxidant capacity (Figure 2). Ultimately, the accumulation of oxidative damage in these compartments may disrupt neuronal integrity, weaken synaptic functions, and contribute to degenerative processes [47].

The potential of AgNPs to induce oxidative stress in rat brain tissue was demonstrated by Skalska et al. [79]. Citrate-stabilized AgNPs of 10 nm were administered orally at 0.2 mg/kg during the experiments. Following 14 days of AgNP exposure, 83.8% and 20% increases were noted for ROS and MDA levels, respectively. Analysis of antioxidant defense parameters revealed a 17% increase in GPx activity while SOD and CAT activities had a negligible change. The GSH/GSSG ratio decreased by 42%. Consequently, these findings indicate that even at low doses, AgNPs contribute to early-stage neurotoxicity by concurrently inducing mitochondrial impairment and ROS accumulation. This combined bioenergetic and redox failure may compromise neuronal integrity.

### 3.3. Neuroinflammation and Glial Activation

AgNP exposure may induce oxidative stress and tissue damage, leading to early activation of immune-responsive glial cells. In this context, microglia and astrocytes are among the first responders to these signals and play key roles in modulating the neuroinflammatory response. Microglia exhibit rapid activation in response to stress signals, adopting a reactive, proinflammatory functional state. Astrocytes are also affected early in this process due to their position between the capillary structure and neuronal network. In response to stress signals, astrocytes can reduce the priority of their metabolic and homeostatic functions and begin to show morphological and functional markers of a reactive phenotype [33]. The simultaneous activation of both cell types mounts a defense response. This activation also creates a more reactive microenvironment. Such a microenvironment can destabilize the synaptic environment and facilitate the spread of inflammatory signals thereby promoting biological conditions favorable to the exacerbation of neuronal damage at later stages [80].

Induction of glial and inflammatory changes due to AgNP exposure in the brain tissue were evaluated by Xu et al. in vivo [81]. Sprague Dawley rats were exposed to AgNPs through an intragastric route at doses of 1 mg/kg and 10 mg/kg. Accumulation of silver in the brain was determined following 14 days of treatment. Histological examinations revealed marked neuronal shrinkage, swelling in astrocytes at the cytoplasmic and end-foot levels and lymphocyte accumulation in the perivascular region. Notable increases in IL-4 levels and a slight increase in IL-6 levels were detected in serum analyses. In addition, 2.77 and 1.45-fold increases were observed for the expression of BBB-associated Claudin-1 and Cadherin-1.

Increasing glial activation leads to a more complex inflammatory signaling network in the neural microenvironment, involving key signaling pathways and molecular regulators. Through the activation of Toll-like receptors and nuclear factor kappa B (NF-κB) pathways, AgNP exposure can increase the transcription level of proinflammatory cytokines such as TNF-α, IL-1β, and IL-6 in microglia. These cytokines can amplify inflammation and increase permeability in the endothelial structure of the BBB. Thus the passage of peripheral immune cells into brain tissue may be facilitated [80]. In this process, astrocytes increase NF-κB-mediated glial fibrillary acidic protein (GFAP) expression and, by stimulating the release of chemokines such as the CCL2/CCR2 axis and CXCL13, may strengthen the migration of microglia and lymphocytes into the region. In addition to these cellular processes, COX-2-mediated prostaglandin E_2_ (PGE_2_) production can further support inflammatory signaling, while decreases in anti-inflammatory molecules, such as IL-10 and TGF-β, sustain ongoing inflammation (Figure 2). Therefore, AgNP exposure leads glial cells to assume an active role in maintaining the inflammatory response [82].

Investigating the cellular response related to this process, Duffy et al. carried out an in vitro study to evaluate how low-dose AgNP exposure triggers the microglial response [83]. In this study, AgNPs with a diameter of 20 ± 4 nm were applied to BV2 microglial cultures at various concentrations, and after short- and medium-term incubation, a significant increase in TNF-α levels was recorded. In a manner accompanying this increase, it was determined that the NF-κB p65 subunit exhibited marked phosphorylation at 30 min and that ROS increased to a significant level at 75 min. Under the same conditions no increase in microglial cell death was observed. Additionally, decreased hypothalamic neuron viability was measured after exposure to conditioned medium from AgNP-stimulated microglia. In summary, these in vivo and in vitro findings suggest that AgNPs induce neuroinflammation via a synergistic interplay involving oxidative stress, NF-κB–mediated glial activation and impaired BBB integrity. This process establishes a self-reinforcing feedback loop, promoting progressive synaptic dysfunction and neuronal loss (Figure 2).

### 3.4. Apoptosis, Autophagy, and Ferroptosis

AgNPs can induce various stress pathways that weaken neuronal cells through the mechanisms discussed above. Cellular defense systems are strained by this environment and become insufficient to maintain intracellular balance. Thus, cell death pathways can be activated including apoptosis and under certain conditions necrosis. Neurons activate clearance mechanisms such as autophagy at an early stage to limit these processes [84].

Apoptosis is an intrinsic pathway triggered by mitochondrial dysfunction and ROS accumulation. In this process, caspase activation may cause the cell to enter an irreversible death program. On the other hand, autophagy is a pathway that enables damaged organelles and proteins to be sequestered within autophagosomes and then degraded through fusion with lysosomes. This clearance mechanism is important for the maintenance of cellular homeostasis and for sustaining its functionality [85,86]. Initially, autophagy is a compensatory response against AgNP-induced oxidative stress and calcium imbalance. However AgNPs can cause lysosomal dysfunction, increased membrane permeability and disruptions in fusion processes. Autophagic flux can slow significantly due to these alterations. Accumulation of markers such as LC3-II and p62 indicates that damaged mitochondria cannot be removed and AgNPs make cellular energy balance more vulnerable. Thus, AgNPs may exacerbate neuronal damage by disrupting the reciprocal relationship between these two cellular processes [82].

Chang et al. investigated AgNP-induced oxidative stress, autophagy, and apoptosis, and assessed how these responses relate to the PI3K/AKT/mTOR signaling axis [87]. For this purpose, HT22 cells derived from mouse hippocampal neurons were exposed to AgNPs at different concentrations for 24 h. A dose dependent reduction in cell viability and a marked increase in ROS levels were detected. TEM analyses revealed autophagosome accumulation, lysosomal damage, and mitochondrial cristae disruption and swelling. These ultrastructural changes were observed at 25–50 µg/mL AgNPs. Consistent with these cellular changes, an increase in LC3-II/I and a decrease in p62 expression are indicative of autophagy activation. Increases in caspase-3 and Bax, a decrease in Bcl-2, and an increase in the Bax/Bcl-2 ratio indicate that mitochondria dependent apoptosis is activated. Furthermore, changes in the phosphorylation status of PI3K/AKT/mTOR pathway proteins were observed. This signaling regulation was reported to play a key role in the progression of both autophagy and apoptosis.

AgNP exposure can create a biochemical environment conducive to ferroptosis, an iron-dependent form of cell death, by increasing iron accumulation in some tissues, accelerating lipid peroxidation and impairing antioxidant defense. In this ferroptotic process, elevated Fe^2+^ levels and MDA accumulation indicate accelerated oxidative degradation of membrane lipids and increased sensitivity of cellular integrity to redox imbalance. Therefore, the oxidative stress induced by AgNP exposure may also activate ferroptotic responses under suitable conditions and in a cell type dependent manner [88,89].

Niu et al. conducted an in vitro study to investigate how AgNP exposure triggers ferroptosis in HT22 hippocampal neurons [90]. In this in vitro study, AgNPs were applied at concentrations of 2–8 µg/mL for 24 h. FerroOrange staining analyses revealed that intracellular ferrous iron (Fe^2+^) levels increased dose-dependently within the 2–8 µg/mL AgNP range, with the most marked increase at 8 µg/mL. Compared with the control group, a 5.85-fold increase in lipid peroxidation and a marked decrease in GPX4 expression were observed; GSH levels decreased to 52.54% of the control. It was shown that ferroptosis-related signaling was activated with increases in ACSL4 and COX-2 levels, and that iron homeostasis was disrupted with increases in TFRC, FTH1, and FTL.

Collectively, these findings indicate that AgNP exposure activates multiple cell death pathways through interconnected stress responses. Specifically, ROS generation and PI3K/AKT/mTOR activation can trigger apoptosis and autophagy while iron imbalance and impaired antioxidant defenses induce ferroptosis. Once activated, these distinct cell death pathways (Figure 2) contribute to progressive neuronal damage. Taken together, AgNP exposure can cause significant influence on the nervous system. AgNPs can reach the CNS through the BBB or alternative pathways and disrupt BBB integrity, allowing particles and ions to enter nervous tissue. Within the CNS, excess amounts of AgNPs can induce the aforementioned neurotoxic mechanisms. These mechanisms generally operate in a cycle, triggering one another and further increasing the neurotoxicity. In developmental neurotoxicity, the effects of these AgNP-induced mechanisms may be more significant [39]. Considering the BBB maturation in the developing brain is incomplete and age specific transport systems are present, AgNPs can enter the brain more readily and remain retained in brain tissue. Moreover, AgNP exposure can disrupt glutamatergic neurotransmission and NMDA-dependent signaling key processes in synaptic plasticity. Such signaling changes can increase the risk of long term synaptic dysfunction in the developing brain. Therefore, evaluating the neurotoxic effects of AgNPs requires an integrated mechanistic approach that considers the various mechanisms underlying neurotoxicity and the specific vulnerabilities of both the adult and developing brain.

## 4. Experimental Evidence: In Vitro and In Vivo Studies

Recent investigations into the neurotoxic potential of AgNPs have revealed various adverse effects of these nanoparticles on the nervous system. They have also highlighted the importance of understanding the key factors influencing this neurotoxic potential and the molecular mechanisms involved. The main molecular mechanisms of the potential neurotoxicity induced by AgNPs and the parameters that may influence this potential have been discussed in the previous sections of this review. Following this overview, in this section, in vitro cell/tissue culture and in vivo animal model studies published over the past five years that investigate the effects of AgNPs on the nervous system in experimental conditions will be presented. Thus, the aim is to evaluate the effects of AgNPs on the nervous system through various experimental models, associating them with nanoparticle properties, molecular mechanisms, and neurotoxic outcomes.

### 4.1. In Vitro Models

Zhai et al. conducted an in vitro study to investigate whether polyvinylpyrrolidone (PVP)-coated AgNPs induce size-dependent neurotoxicity in human SH-SY5Y neuroblastoma cells through ferritinophagy-mediated oxidative stress and disruption of iron metabolism [91]. For this purpose, SH-SY5Y cells were exposed to 5 nm (Ag-5) and 50 nm (Ag-50) PVP-coated AgNPs at concentrations of 40 and 60 µg/mL for 24 h, and cell viability was evaluated using the CCK-8 assay. It was reported that these exposures reduced cell proliferation in a dose- and size-dependent manner, with the lowest viability observed in the 60 µg/mL Ag-5 group at approximately 70.7%, and that Ag-5 exhibited a more pronounced cytotoxic effect than Ag-50 at the same dose. BACE1 protein and the gene levels of *APP* and *ADAM10* were analyzed to monitor changes in the APP amyloid pathway. These analyses showed significant increases in BACE1 protein and *APP* mRNA levels, whereas ADAM10 expression exhibited marked decreases in most groups. The protein levels associated with ferritinophagy and the autophagic response (NCOA4, FTH1, Atg7, LC3B-II/I, and p62) were measured. The observation of increases in most of these proteins and accumulation of p62 suggested that ferritinophagy was activated but that autophagic flux was partially impaired at a late stage. In parallel, intracellular iron content and expression of the iron transport-related genes *DMT1* and *FPN1* were assessed. It was shown that exposure to AgNPs generally led to significant increases in iron levels and upregulation of *DMT1* and *FPN1*. Additionally, the highest increase in *DMT1* gene expression was recorded in the 40 µg/mL Ag-50 group, at about 1.42-fold compared to the control. The oxidative stress response was examined using GPX-4 protein level and MDA, an indicator of lipid peroxidation; with AgNP exposure, a decrease in GPX-4 and a more pronounced increase in MDA, particularly with Ag-5 exposure, were detected. Overall, the findings from this in vitro study indicate that PVP-coated AgNPs induce marked and size-dependent neurotoxicity in SH-SY5Y cells by accelerating the APP amyloid process through ferritinophagy-related iron accumulation and oxidative stress.

Zhang et al. investigated the size- and form-dependent neurotoxic effects of PVP-coated AgNPs and Ag^+^ ions on mature and developing primary rat cerebral cortical neurons [92]. In this in vitro study, primary cortical neuron cultures were exposed to PVP-coated 20 nm (AgNPs-20) and 70 nm (AgNPs-70) AgNPs and to Ag^+^ (AgNO_3_) for 24 h at 0.01–40 µg/mL in mature cells and for 7 days at 0.01–5 µg/mL in developing cells, and cell viability was evaluated using the alamarBlue assay. In the 24 h exposures, all three silver forms reduced cell viability in a concentration-dependent manner, and the EC_50_ values were reported as 2.86 ± 0.12 µg/mL for AgNO_3_, 6.61 ± 0.28 µg/mL for AgNPs-20, and 38.4 ± 1.61 µg/mL for AgNPs-70. During this period, the highest toxicity level was observed with AgNO_3_ exposure, followed by AgNPs-20, and the lowest with AgNPs-70. In the 7-day exposures, decreases in the EC_50_ values to 1.08 ± 0.40, 2.02 ± 0.07, and 1.22 ± 0.04 µg/mL for AgNPs-20, AgNPs-70, and AgNO_3_, respectively, a marked increase in cytotoxicity, and a decline in the no-effect level to approximately 0.5 µg/mL were recorded. Changes in neuronal morphology were examined by TuJ1 immunostaining; in high-dose short-term exposures, particularly with AgNPs-20 and AgNO_3_, a more granular cytoskeletal structure, broken neurites, and disruption of network integrity were observed. At lower doses and in the 7-day exposures, thinning of filaments and a fragmented network appearance were detected for all three silver forms. In addition, intracellular silver accumulation at 0.5 µg/mL was measured by ICP-MS, and the highest accumulation was found in the AgNPs-20 group, with lower levels in the AgNPs-70 and AgNO_3_ groups, respectively. To evaluate the neuronal functional response, extracellular dopamine levels in surviving cells were analyzed by ELISA; in mature neurons, AgNP exposures in the 0.01–0.5 µg/mL range reduced dopamine release in a concentration-dependent manner, and this effect was more pronounced with AgNPs-20. Exposure to 0.05 µg/mL AgNO_3_ under comparable conditions did not significantly alter dopamine levels, whereas in developing neurons, 7-day exposures to 0.01–0.5 µg/mL AgNPs led to similar decreases in dopamine release, and these cells were determined as particularly sensitive to AgNPs-20. Ultimately, these findings demonstrate that PVP-coated AgNPs induce marked neurotoxicity in primary cortical neurons through size and form specific cytotoxicity, cytoskeletal degeneration, intracellular silver accumulation, and suppression of dopamine release. When integrated with the data presented by Zhai et al., the neurotoxic potential of PVP-coated AgNPs is directly related to particle size and exposure dose. In both neuronal models, neurotoxic damage intensified as particle size decreased and dose increased. However, these two studies focused on different aspects of toxicity. Zhai et al. aimed to elucidate ferritinophagy-mediated iron accumulation, oxidative stress, and APP amyloid pathway alterations as mechanisms underlying cellular damage. Zhang et al. demonstrated cytoskeletal disruption and suppression of dopamine release, focusing predominantly on structural and functional outcomes. Furthermore, this study underscored the increased susceptibility of developing neurons to prolonged exposure and the potentially greater toxicity of free Ag^+^ ions relative to the particulate form. These two studies indicate a higher neurotoxic potential for small PVP-coated AgNPs, which presents differently across varied neuronal models.

As another example, Pavičić et al. investigated the potential for neurodevelopmental and neurodegenerative toxicity of AgNPs stabilized with different coating agents in embryonic murine neural stem cells (mNSCs) [93]. In this in vitro study, AgNPs were synthesized by chemical reduction of silver nitrate, and subsequently coated with sodium bis(2-ethylhexyl) sulfosuccinate (AOT, negative), cetyltrimethylammonium bromide (CTAB, positive), polyvinylpyrrolidone (PVP, nominally neutral), poly-L-lysine (PLL, positive), and bovine serum albumin (BSA, negative), and AgNO_3_ was used as a source of Ag^+^ for comparison. Characterization studies revealed that the main population of the particles was below 15 nm, mostly spherical in shape, and remained stable in the culture medium through protein corona formation, and that under exposure conditions only a very small fraction (≈0.2%) of the total silver could dissolve as Ag^+^. A concentration-dependent decrease in cell viability was observed for all coating types after 24 h exposures to AgNPs and AgNO_3_ in the CCK-8 assay, with a marked loss beginning at around 5 mg Ag/L. Cytotoxicity analyses performed under the same conditions showed that AgNPs caused marked cytotoxicity starting from about 1 mg Ag/L, that toxicity was highest in CTAB-coated AgNPs and lowest in BSA-coated AgNPs, and that the toxic threshold for ionic Ag appeared at lower concentrations. Additionally, it was determined that in AOT-, PLL-, and BSA-coated AgNP exposures cell death occurred predominantly through apoptosis, that necrosis was more predominant with CTAB-coated AgNPs, and that in PVP-coated AgNPs the apoptotic response prevailing at low doses shifted toward necrosis at higher doses. Based on mitochondrial and plasma membrane potential analyses, mitochondrial changes remained limited under CTAB-AgNP exposure, whereas early mitochondrial hyperpolarization and plasma membrane hyperpolarization suppressing proliferation were detected in exposures to AOT-, PVP-, PLL-, and BSA-coated AgNPs and to Ag^+^. These findings suggested a functional response consistent with apoptosis in the cells. In the evaluations of oxidative stress and antioxidant response, it was found that all AgNP types increased ROS, and that the increase in superoxide production was more pronounced, particularly in PVP- and AOT-coated AgNPs and Ag^+^ exposures. Additionally, in most AgNP groups, the increase in intracellular GSH level was determined to reflect a compensatory antioxidant response, whereas in Ag^+^ exposure the decrease in GSH and the impairments in SOD and GPx activities indicated a more severe oxidative damage profile. Gene expression analyses showed that, particularly in PVP-, AOT-, and PLL-coated AgNP exposures, changes in *Nestin* and *Doublecortin* levels affected the neural stem cell phenotype and early neuronal differentiation, and that CTAB-, PVP- and AOT-coated AgNPs induced a marked IL-6-mediated inflammatory response. Moreover, in the alkaline comet assay, it was observed that all AgNP types increased primary DNA damage, that this effect was more pronounced in positively charged PLL- and CTAB-coated AgNPs, and that under the same conditions no significant genotoxicity was observed in Ag^+^ exposures. Taken together, these findings indicate that AgNPs stabilized with different coating agents exhibit a marked potential for neurodevelopmental and neurodegenerative neurotoxicity in mNSCs, which cannot be explained by Ag^+^ release alone and is mediated by loss of cell viability, disruption of the apoptosis/necrosis balance, oxidative stress, inflammation, gene expression changes and DNA damage. In this respect, the study differs from the first two investigations highlighting the prominence of particle size under the same coating. Even for AgNPs within a similar size range, the neurotoxic profile is observed to vary markedly depending on the surface coating and charge. Consistent with previous investigations, a reduction in cell viability and an oxidative stress response were also observed in the present model. However, the effects on the apoptosis/necrosis balance, inflammatory response, DNA damage and neural differentiation revealed a more comprehensive damage profile in this study. Therefore, AgNP neurotoxicity emerges as a multifaceted process shaped by surface properties as well as particle size.

In another in vitro study, Chang et al. aimed to elucidate the neurotoxicity induced by PVP-coated AgNPs in mouse hippocampal HT22 neurons, particularly in terms of ROS–Drp1-mediated mechanisms underlying mitochondrial damage and apoptosis [94]. For this purpose, PVP-coated AgNPs with a core size of 23.53 ± 4.79 nm were characterized, and HT22 cells were then exposed for 24 h to increasing concentrations of AgNPs or AgNO_3_. Following these exposures, it was found in the CCK-8 assay that cell viability decreased in a concentration-dependent manner and that the IC_50_ values for AgNPs and AgNO_3_ were 10.8 and 0.62 µg/mL, respectively. In ICP-MS analyses, it was determined that at an 8 µg/mL AgNP dose only approximately 3% of the silver taken up per cell was in the Ag^+^ form and that the level of free Ag^+^ in the culture medium remained very low. These findings suggest that the overall cytotoxic effect could be largely attributed to the particulate form. TEM and Mitotracker staining showed that mitochondrial network integrity was disrupted by exposure to 8 µg/mL AgNPs and that at this dose the number of fragmented mitochondria increased by approximately 3.76-fold, the aspect ratio decreased by about 40.6%, and the form factor increased by approximately 1.9-fold. In contrast, no marked change in mitochondrial morphology was reported at comparable AgNO_3_ concentrations. In functional assessments, exposure to 8 µg/mL AgNPs was observed to increase intracellular ROS levels by approximately 3.82-fold compared with the control, to decrease the mitochondrial membrane potential, and to reduce ATP levels by 32.6%. In contrast, AgNO_3_ exposures were found not to alter these parameters to a significant extent. Analyses of proteins related to mitochondrial dynamics showed that AgNP exposure increased the expression of Drp1 and p-Drp1(Ser616) and enhanced the translocation of Drp1 to mitochondria. At an 8 µg/mL AgNP dose, the p-Drp1 (Ser616) level increased by approximately 2.27 fold compared with the control group. Consistent with disrupted mitochondrial fission/fusion dynamics, changes in Fis1 and OPA1 levels were observed. In parallel, increases in Bax and cytochrome c levels, a decrease in Bcl-2, and an increase in cleaved caspase-3 were detected. These apoptosis related changes suggest a more pronounced activation of mitochondria-dependent apoptosis. To further evaluate the roles of ROS and Drp1 in these effects, antioxidant pretreatment with *N*-acetyl-L-cysteine (NAC) and pharmacological inhibition with the Drp1 inhibitor Mdivi-1 were applied. Pretreatment with NAC was observed to suppress the AgNP-induced increase in ROS, to partially restore the mitochondrial membrane potential and ATP levels, and to attenuate both Drp1 activation and the changes in apoptosis markers. By contrast, Mdivi-1 treatment was determined to reduce mitochondrial fission and apoptosis, whereas ROS levels were not significantly altered. As a result, the findings from this in vitro study provide evidence that AgNP-induced neurotoxicity in HT22 cells, largely independently of free Ag^+^, leads to mitochondria-dependent apoptosis progressing through increased ROS and Drp1-mediated excessive mitochondrial fission.

In a further example, Shang et al. conducted an in vitro study to evaluate the neurotoxic potential of AgNPs in murine BV2 microglial cells through inflammatory phenotypic polarization and the autophagy–lysosome axis [95]. In this study, BV2 microglial cells were exposed to PVP-coated AgNPs with an average diameter of 23.44 ± 4.92 nm at concentrations ranging from 1.25 to 200 µg/mL for 12, 24, and 48 h, and cell viability was assessed using the CCK-8 assay. At a dose of 5 µg/mL, cell viability was measured at approximately 95.39% and 94.68% after 12 and 24 h of exposure, respectively, and at 78.13% at 48 h. Therefore, 5 µg/mL was selected as a non-cytotoxic concentration for exposures of up to 24 h. Under the same conditions, gene expression and cytokine analyses revealed significant increases in the mRNA levels of Iba-1, MCP-1, IL-1β, TNF-α, and NF-κB associated with the M1 phenotype. In contrast, decreases in the expression of IL-10 and TGF-β associated with the M2 phenotype and time-dependent increases in the secretion of TNF-α and IL-6 in the supernatant were detected. In addition, intracellular AgNP uptake was examined by flow cytometry, and it was reported that the side scatter signal generally increased over a 2–24 h period at a 5 µg/mL exposure and showed a slight decrease at 24 h. Consistent with these findings, flow cytometry and Western blot analyses showed that the proportion of iNOS-positive M1 cells and iNOS protein levels increased over time, whereas the proportion of CD206-positive M2 cells and CD206 protein levels decreased. Additionally, increases in NF-κB and IL-1β protein levels were observed to support inflammatory M1 polarization. At the ultrastructural level, TEM examinations revealed that control cells maintained normal nuclear and mitochondrial morphology and contained only a limited number of autophagic vesicles. In contrast, after 24 h of exposure to 5 µg/mL AgNPs, a large accumulation of autophagosomes and autolysosomes, as well as dense autophagic vacuoles containing partially degraded cytoplasmic material within these structures, was detected. Under the same experimental conditions, time-dependent increase in LC3-II and Beclin1 protein levels, accumulation of p62/SQSTM1, and decreases in the levels of the lysosomal markers, LAMP2, LAMP1, and CTSB were recorded. This protein pattern was found to be consistent with a blockade of autophagic flux at the lysosomal stage and with weakened lysosomal function. To evaluate the functional role of autophagy, BV2 cells were pretreated with the autophagy inhibitor 3-MA and subsequently exposed to 5 µg/mL AgNPs for 24 h. Under these conditions, an additional decrease in LC3-II, LAMP1, and CTSB levels was reported. Furthermore, iNOS expression and TNF-α and IL-1β mRNA levels were observed to be higher than with AgNP exposure alone, whereas CD206, IL-10, and TGF-β expression levels were more markedly suppressed. In conclusion, the findings from this in vitro study indicate that AgNPs, at non-cytotoxic concentrations, disrupt autophagy and lysosomal function in BV2 microglial cells, thereby triggering a predominantly M1 inflammatory phenotype and contributing to a microglia-mediated mechanism of neurotoxicity. When evaluated together with the HT22 model in the previous study, AgNPs within a similar size range and identical PVP coating induce marked cellular stress and dysfunction in both models. However, the predominant neurotoxic mechanisms vary across cell types. Chang et al. revealed that the damage process in HT22 cells proceeds through elevated ROS and Drp1-mediated excessive mitochondrial fission and is associated with mitochondria-dependent apoptosis. Shang et al. linked lysosomal dysfunction and impaired autophagy flux in BV2 microglia predominantly to M1 inflammatory polarization. All these in vitro studies demonstrate that AgNP neurotoxicity is a multidimensional process shaped by the interplay of physicochemical properties, exposure conditions, and the target cell type, varying at both mechanistic and outcome levels.

### 4.2. In Vivo Models

Niu et al. investigated the neurobehavioral changes induced by AgNP exposure, silver (Ag) accumulation in the brain, and ferroptosis-related mechanisms in male ICR mice [96]. In this in vivo study, suspensions of PVP-coated AgNPs with an average diameter of 18.39 ± 5.57 nm at doses of 5 and 50 mg/kg, and 0.4 mg/kg AgNO_3_ to represent free Ag^+^ ions, were administered intranasally once daily for 28 days. Following a 28-day recovery period after exposure, Ag accumulation in blood, brain regions, and peripheral organs and behavioral, histopathological, and biochemical parameters were evaluated. ICP-MS analyses showed that the highest Ag accumulation occurred in the lungs and kidneys, particularly in the 50 mg/kg AgNP group, and that Ag also reached the brain. Within the brain, it was indicated that Ag accumulation was not evenly distributed, that Ag levels in the hippocampus, which is related to learning and memory, increased to approximately 1.64–3.75 times the whole-brain average, and that the hippocampus was the main accumulation region in the brain. In addition, it was reported that during the recovery period Ag levels in the olfactory bulb decreased markedly, particularly in the high-dose AgNP and AgNO_3_ groups, whereas Ag elimination in the brain and hippocampus remained limited and residual Ag levels in these regions remained significantly high. In the open field test (OFT), no marked change was detected in locomotor activity and anxiety-related parameters. By contrast, the Morris water maze (MWM) test showed that, particularly in the 50 mg/kg AgNP group, the escape latency was prolonged, the time spent in the platform quadrant and the number of platform crossings were reduced, and this spatial memory impairment was maintained in the repeated tests during the recovery period. Additionally, significant negative correlations were observed between Ag levels in the whole brain and hippocampus and spatial memory indices, such as the time spent in the target quadrant and the number of platform crossings, and spatial memory performance worsened as Ag accumulation in these regions increased. Nissl staining analyses revealed that, in hippocampal CA3 neurons exposed to AgNPs, cellular integrity was disrupted, cytoplasmic atrophy and vacuolization were increased, and nuclear morphology was markedly altered. In contrast, neuronal degeneration of comparable severity was not observed in the AgNO_3_ group. In TEM analyses, ferroptosis-specific mitochondrial ultrastructural features, such as alterations in mitochondrial volume, loss of cristae, and increased membrane density, were identified in hippocampal neurons exposed to AgNPs and AgNO_3_. In the assessment of lipid peroxidation and antioxidant responses in brain tissue, the decrease in GSH levels, particularly in the 50 mg/kg AgNP and AgNO_3_ groups, and the persistently elevated MDA levels throughout the exposure and recovery periods indicated sustained oxidative stress and lipid peroxidation. Western blot and immunohistochemical analyses revealed that exposure to 50 mg/kg AgNPs and AgNO_3_ suppressed NRF2 and HO-1 expression, while p53 expression was upregulated in the AgNP groups but downregulated in the AgNO_3_ group. These analyses also showed reduced levels of iron storage proteins such as TFRC, FTH1, and FTL, as well as the ferroptosis-suppressing proteins GPX4 and SLC7A11. In contrast, it was found that ACSL4 and COX2 expression was increased, whereas SLC7A11 staining was markedly reduced in the hippocampal CA1/CA3 regions. These protein alterations strongly support the activation of the ferroptosis pathway. Moreover, the fact that changes in ferroptosis markers and lipid peroxidation in the 0.4 mg/kg AgNO_3_ exposure group were similar in magnitude to those in the high-dose AgNP group suggested that free Ag^+^ might be a stronger inducer of ferroptosis than AgNPs. Taken together, the findings from this in vivo study indicate that repeated intranasal exposure to PVP-coated AgNPs and AgNO_3_ causes persistent hippocampal Ag accumulation and ferroptosis-related damage, leading to lasting spatial learning and memory impairments in mice.

In another in vivo study conducted in the same male ICR mouse model, Chang et al., aimed to evaluate the neurotoxicity of AgNPs in the hippocampus associated with mitochondrial damage, mitophagy/mitochondrial biogenesis impairment, and synaptic degeneration [97]. For this purpose, suspensions of PVP-coated AgNPs with a core diameter of 23.53 ± 4.79 nm at doses of 12 and 120 mg/kg, 5 mg/kg AgNO_3_ solutions for comparison, and 5% glucose as the control were administered intravenously via the tail vein to the mice on days 1, 4, 7, and 10. On days 7 and 14 after the last injection, brain and hippocampal tissues were collected for behavioral, histopathological, ultrastructural, ICP-MS, immunohistochemical, and Western blot analyses. It was reported that body weight decreased significantly on post-injection day 7, whereas on day 14 it did not differ significantly from the control group, and that brain organ coefficients did not show significant changes at either time point. In the MWM test, it was observed that AgNP exposure, particularly at the 120 mg/kg dose, prolonged the time required to find the platform and that the escape latency remained higher than in the control group throughout the training period. This was interpreted to reflect impairment of spatial learning and memory functions in the mice. Histopathological examinations revealed, in the 120 mg/kg AgNP group, disruption of neuronal integrity, cytoplasmic shrinkage, vacuolization, and marked changes in nuclear morphology in the hippocampal CA3 region. In contrast, degeneration of similar severity was not observed in the cortex or in the AgNO_3_ group. Based on Nissl staining, disruption of neuronal integrity in hippocampal CA3 neurons, cytoplasmic shrinkage and vacuolation, and loss of distinct nuclear morphology were determined, and these degenerative changes persisted on post-injection day 14. Additionally, similar degenerative changes were not detected in the AgNO_3_ group. In ICP-MS analyses, Ag accumulation in the hippocampus was measured to increase significantly with AgNP exposure and to reach 193.65 and 171.76 ng/g body weight in the 120 mg/kg AgNP group on post-injection days 7 and 14, respectively. TEM examinations showed that after exposure to 120 mg/kg AgNPs, accumulation of AgNPs in the cytoplasm of hippocampal neurons was associated with rupture of the outer mitochondrial membrane, loss of cristae, and vacuolization. Immunohistochemical and Western blot analyses revealed that exposure to 12–120 mg/kg AgNPs increased PINK1 and Parkin protein expression and decreasing PGC-1α levels in hippocampal neurons, whereas AgNO_3_ exposure increased only PINK1 expression and decreased PGC-1α levels. Moreover, AgNP exposure was reported to increase synaptophysin and APP protein levels and to decrease PSD95 and MAP2 levels in the hippocampus. In ultrastructural and immunofluorescence examinations, blurring of the hippocampal synaptic cleft, thickening of the postsynaptic density, sparse branching of neuronal dendrites, and reduced MAP2 immunoreactivity were observed following exposure to 120 mg/kg AgNPs. Although the pattern of protein expression under AgNO_3_ exposure was similar to that with AgNPs, no marked histopathological or ultrastructural abnormalities were detected. Overall, the findings from this in vivo study provide evidence that intravenous exposure to PVP-coated AgNPs impairs spatial learning and memory in male ICR mice through Ag accumulation, mitochondrial damage, disturbed mitophagy/mitochondrial biogenesis, and synaptic degeneration in the hippocampus, and that this neurotoxic effect is largely specific to the particulate form. When these two studies are evaluated together, PVP-coated AgNPs used in the same ICR mouse model are observed to consistently target the hippocampus. Despite differences in particle size, exposure route and dose, impairments in spatial learning and memory were reported in both studies. At the tissue level, both studies associated these neurobehavioral impairments with hippocampal Ag accumulation and neuronal damage, particularly in the CA3 region. Niu et al. primarily emphasized ferroptosis-related oxidative and lipid damage, whereas Chang et al. highlighted mitochondrial quality control impairment and synaptic degeneration. This suggests that AgNP neurotoxicity may develop through multiple interrelated mechanisms in the same target tissue.

Dziendzikowska et al. evaluated how AgNPs with different surface coatings affected hippocampal oxidative stress pathways and neurosteroid metabolism in male Wistar rats [98]. In this in vivo study, three experimental groups were exposed to AgNPs coated with BSA, PEG, or citrate, and another group was exposed to AgNO_3_ as a source of Ag^+^, with all silver forms administered by oral gavage at a dose of 0.5 mg/kg body weight, while the control group received the same volume of water. These treatments were continued for 28 days, and after 1 week of spatial memory training, hippocampal tissues were collected for biochemical and gene expression analyses. In the hippocampus, the concentrations of pregnenolone, progesterone, 17α-progesterone, allopregnanolone, DHEA, DHEAS, androstenedione, 17β-estradiol, testosterone, and dihydrotestosterone were measured by LC-MS/MS, and coating- and silver form-dependent changes were detected. Progesterone levels were observed to be significantly decreased in the BSA-AgNP, PEG-AgNP, and Ag^+^ groups, and 17α-progesterone levels were significantly decreased in the Cit-AgNP, BSA-AgNP, and Ag^+^ groups compared with the control group. In contrast, allopregnanolone levels were found to be highest in the BSA-AgNP group, with significantly higher values than in the control group, and to be markedly increased in the BSA- and PEG-AgNP exposure groups compared with the Cit-AgNP and Ag^+^ groups. It was reported that the most pronounced increase in DHEA and DHEAS levels occurred in the Ag^+^ group and that the concentrations of androstenedione and 17β-estradiol also increased predominantly in association with Ag^+^ exposure and partly with the BSA-AgNP group. Additionally, that testosterone levels in the Ag^+^ group were lower than in the BSA- and Cit-AgNP groups, whereas DHT levels in the Ag^+^ and Cit-AgNP groups were higher than in the control and PEG-AgNP groups, was determined. In addition to these changes in the neurosteroid profile, hippocampal antioxidant enzyme activities and oxidative status markers were also analyzed. That SOD activity in the BSA- and PEG-AgNP groups increased compared with the control, Cit-AgNP, and Ag^+^ groups, whereas GPx activity was elevated in the Cit- and BSA-AgNP and Ag^+^ exposure groups, was observed. These findings showed that total antioxidant capacity was decreased in the BSA-AgNP group compared with the other Ag-exposed groups and that TBARS levels were increased in the Cit- and BSA-AgNP groups relative to PEG-AgNP and Ag^+^ exposures. qPCR analyses revealed marked changes in the hippocampal expression of some genes involved in antioxidant defense and neurosteroidogenesis. With respect to the antioxidant response, it was recorded that *Cat* expression was significantly decreased in the PEG-AgNP and Ag^+^ groups, whereas *Gsr* expression was significantly decreased only in the Ag^+^ group. In contrast, no marked changes were observed in *Sod1*, *Sod2*, *Gpx1*, or *Hmox1* expression. In terms of genes related to steroidogenesis and steroid signaling, Ag^+^ exposure was found to reduce the expression of *Star*, *Hsd3b3*, *Hsd17b10*, *Hsd11b2*, and *Cyp21a1*; Cit-AgNP exposure reduced the expression of *Hsd17b1*; and BSA-AgNP exposure reduced the expression of *Hsd17b10* and *Hsd11b2*. Moreover, a general downregulation of the steroid receptor genes *Ar*, *Er1*, and *Er2* was detected in the AgNP and Ag^+^ groups, and these steroid receptor-related changes were consistent with the disruption of the neurosteroid profile. In conclusion, these findings indicate that 28-day oral exposure to AgNPs stabilized with different coating agents and Ag^+^ induces coating material- and silver form-dependent oxidative stress and neurosteroid imbalances in the rat hippocampus, and that these changes may contribute to Ag-induced neurotoxicity. Similar to the first two studies, the present findings confirm that the hippocampus is a region sensitive to AgNP exposure. However, the animal model, administration route, and dose differ in this study. Furthermore, the neurotoxic effects of AgNPs with various surface coatings were compared instead of using a uniform PVP coating. This study focused on biochemical changes rather than the structural and behavioral impairments prominent in previous studies. Specifically, oxidative stress markers, antioxidant defense response and neurosteroid metabolism were assessed in a coating type-dependent manner within this scope. These molecular findings offer a complementary perspective on AgNP neurotoxicity at a biochemical level.

Yan et al. investigated the neurotoxic effects of AgNPs in zebrafish embryos and larvae and how the protein corona formed by BSA modulates this neurotoxicity [99]. For this purpose, non-transgenic AB wild-type zebrafish embryos and transgenic zebrafish embryos were exposed to control, 50 mg/L AgNPs, 50 mg/L AgNPs + 5 mg/L BSA, or 2.5 mg/L Ag^+^ + 5 mg/L BSA. During this process, basic developmental parameters were assessed between 48 and 120 h post fertilization (hpf); heart, CNS and motor neuron development were evaluated at 96 hpf, and locomotor behavior, neurotransmitter levels and neurodevelopment-related gene expression at 120 hpf. AgNP exposure was observed not to exert significant effects on hatching rate, heart rate, malformation rate, or body length, whereas at 120 hpf it reduced swimming distance and speed by 20–45% during light/dark cycles, resulting in a marked inhibition of locomotor activity. Consistent with these behavioral findings, AgNP exposure caused cardiac developmental abnormalities in the Tg(cmlc2:EGFP) line by increasing cardiac fluorescence intensity and, in the Tg(HuC:EGFP) and Tg(hb9:EGFP) lines, it caused delayed neurogenesis and axonal damage by decreasing neuronal and motor neuron fluorescence. At the transcriptional level in non-transgenic larvae, the expression of *elavl3*, *ngn1*, and *nestin* was significantly suppressed by AgNP exposure, whereas the expression of *gfap* and *gap43* was upregulated, and no marked changes were detected in *syn2a* or *mbp* expression. These gene expression changes are indicative of impaired neuronal differentiation, altered axonal growth and inadequate CNS development. Neurotransmitter analyses showed that AgNP exposure reduced acetylcholine (ACh) levels in larvae by 35% compared with the control, and suppressed the expression of the cholinergic system-related genes *chat*, *vacht* and *ache* by 26%, 60%, and 81%, respectively. By contrast, dopamine (DA) content was not significantly altered in any group; however, the expression levels of th and mao were decreased by 22% and 24%, respectively, and no marked change was measured in dat expression. To elucidate the role of BSA, protein corona characterization was performed. BSA coating was found to increase the hydrodynamic diameter of AgNPs from 13 nm to approximately 31 nm and to shift the zeta potential from −29 mV to −43 mV, thereby increasing particle size and making the surface charge more negative. Consistent with these physicochemical findings, it was observed that in the presence of BSA the disruptive effects of AgNPs on locomotor behavior, neurodevelopment-related gene expression and cholinergic/dopaminergic signaling were largely attenuated and that swimming performance, ACh levels and most neurodevelopmental/neurotransmitter-related gene responses approached control levels. Additionally, in the BSA-Ag^+^ group, locomotor inhibition was reported to persist, whereas neurodevelopmental and neurotransmitter-related gene expression showed a recovery trend similar to, but weaker than, that in the BSA-AgNP group. As a result, these findings provide evidence that AgNPs present in environmental settings may lead to neurotoxicity in zebrafish larvae by disrupting cardiac development, central and peripheral nervous system development, and cholinergic neurotransmission, but that this neurotoxic effect may be substantially attenuated by the protein corona formed by natural organic matter such as BSA.

Using *Caenorhabditis elegans* as an in vivo model, Zhang et al. investigated the neurobehavioral and neuronal effects of prolonged exposure to PVP-coated and uncoated AgNPs [98]. In this study, synchronized L1-stage wild-type and GFP-expressing transgenic *C. elegans* strains were exposed to 20 nm AgNP or 20 nm PVP-coated AgNP suspensions at concentrations of 0.01–10 mg/L from the L1 stage to adult day 1 (48 h) or adult day 4 (6 days). Subsequently, lifespan, locomotor activity, learning and memory, neuronal morphology and neurotransmitter receptor gene expression were evaluated. Lifespan analyses showed that both forms of AgNP significantly reduced nematode survival in a dose- and time-dependent manner and that this effect, particularly under chronic exposure at concentrations ≥ 0.1 mg/L, was more pronounced in the PVP-AgNP group. In contrast, no significant change in lifespan was detected at 0.01 mg/L. In locomotor behavior tests, head thrashes, body bends, pharyngeal pumping frequency, and defecation interval were reported to be significantly impaired by both forms of AgNP at 1–10 mg/L, particularly after 6-day exposure. In addition, PVP-AgNP exposure induced earlier and more pronounced impairment in these parameters than uncoated AgNPs, and the defecation cycle was prolonged even at 0.1 mg/L PVP-AgNP. Learning and memory performance were assessed using chemotaxis and thermotaxis tests. In chemotaxis tests, the chemotaxis index was found to shift from negative to positive with increasing dose and exposure duration, indicating impaired associative learning. In thermotaxis tests, it was determined that the proportion of animals performing isothermal tracking at 20 °C was reduced and that this proportion decreased by approximately 42% under chronic exposure to 0.1 mg/L PVP-AgNPs. At the neuronal level, fluorescence imaging of GFP-expressing neuronal subtypes showed that dopaminergic and GABAergic neurons were particularly susceptible to AgNP-induced damage. Both forms of AgNP at 1–10 mg/L were reported to significantly reduce GFP fluorescence intensity in these neurons, and under PVP-AgNP exposure this reduction was accompanied by fragmented dendrites and neuronal loss. In contrast, no significant changes were detected in glutamatergic neurons, whereas GFP fluorescence in cholinergic neurons decreased mainly after exposure to 1–10 mg/L PVP-AgNP and 10 mg/L uncoated AgNPs. These results suggest that distinct neuronal subtypes exhibit different susceptibilities to AgNP exposure. Consistent with these results, qRT-PCR analyses at the transcriptional level revealed that low-dose PVP-AgNP exposure increased the expression of several neurotransmitter receptor genes associated with the GABAergic (*gab-1*, *ggr-1*) and dopaminergic (*dop-1*) systems. At higher PVP-AgNP doses, however, a broad suppression of *ggr-2*, *ggr-3* and various dopamine receptor genes was identified. Moreover, under exposure to uncoated AgNPs, the extent and magnitude of changes in these receptor genes were noted to remain more limited and weaker. Ultimately, these findings demonstrated that AgNPs induce dose-, form- and time-dependent neurobehavioral and neuronal toxicity in *C. elegans* by impairing locomotor activity, shortening lifespan, and causing damage in various neuronal subtypes. Unlike rodent models, Yan et al. and Zhang et al. used alternative in vivo systems such as zebrafish embryos/larvae and *C. elegans.* In both models, AgNP exposure caused disruptive effects on locomotor behavior and neuronal function. However, the mechanistic focus and toxicity severity varied depending on surface properties and model type. In the zebrafish model, the BSA-mediated protein corona was reported to attenuate the neurotoxic effects. Even so, the damage was still found to mainly affect neurodevelopmental processes and the cholinergic system. In the *C. elegans* system, PVP coating increased toxicity and specific neuronal subtypes were more susceptible to AgNP induced damage. All these in vivo studies show that AgNP neurotoxicity can cause neurotoxic effects at cellular, structural and functional levels in different biological models depending on physicochemical properties and exposure conditions.

Taken all together, the above examples of in vitro and in vivo studies and those summarized in Table 1 support the view that AgNPs possess a considerable neurotoxic potential across diverse experimental models. Notably, these studies point to recurring neurotoxic patterns across different models rather than independent findings. Numerous studies have shown that AgNP exposure reduces neuronal and glial cell viability and disrupts neuronal morphology and connectivity. These findings are frequently reported together with various mechanisms underlying neurotoxicity. Beyond these cellular-level events, existing studies also reveal structural abnormalities including neurite and synaptic damage, myelin disruption, and BBB alterations. In vivo findings suggest that this combined cellular and structural damage may be associated with adverse outcomes such as neurotransmitter imbalances, neurodevelopmental impairments and deficits in learning, memory and locomotor function. Thus, despite different experimental designs and exposure conditions, current data point to a recurring pattern of neurotoxicity after AgNP exposure at cellular, structural and functional levels. To complement this discussion, Table 1 provides a categorized summary of further representative in vitro and in vivo studies, with particular emphasis on recent studies published after 2020. Nevertheless, the current experimental evidence remains insufficient to fully define safe exposure limits, critical windows of susceptibility and the long-term reversibility of AgNP-induced neurotoxicity. In particular, the wide range of particle sizes, shapes, surface chemistries and protein corona profiles, together with differing exposure routes and doses, complicates cross-study comparison and delays the development of robust neurotoxicity risk assessment for AgNPs.

## 5. Conclusions and Future Perspectives

AgNPs, owing to their rapidly expanding fields of use and their increasingly widespread application in biomedical settings, require careful evaluation of the potential risks they may pose to the CNS. In this review, the neurotoxic effects of AgNPs have been evaluated in relation to physicochemical and exposure-related parameters such as particle size, shape, surface chemistry, protein corona, dose, route of exposure, and duration of exposure. Subsequently, the primary neurotoxic mechanisms, ranging from barrier impairment to diverse intracellular stress and regulated death mechanisms, have been discussed. Additionally, experimental findings obtained from various recent in vitro and in vivo models in this field have been presented. The available evidence demonstrates AgNPs can reach the brain under appropriate conditions and exert adverse effects on both neuronal structure and neurobehavioral functions through potential mechanisms of action. Despite these findings, the current experimental data on AgNP-induced neurotoxicity have several important limitations.

One of these limitations is that the available data are predominantly focused on specific experimental models. Most in vitro studies focus on a few standard neuronal and glial cell lines and basic BBB models. However, studies using human-derived neural cells, three-dimensional brain organoids, and advanced microphysiological BBB systems remain markedly limited. Most findings at the in vivo level are obtained from mouse and rat models, while systematic evaluations conducted in large mammalian species that are structurally and functionally closer to the human brain, such as minipigs, remain extremely limited. Moreover, clinical or epidemiological data that quantitatively and well characterize AgNP exposure and directly relate it to neurobehavioral or neurocognitive outcomes are nearly absent. This insufficiency creates substantial uncertainty regarding the extent to which these experimental findings can be generalized to human health.

Another limitation is that current studies are highly variable in terms of design characteristics and evaluation criteria. In different studies evaluating the neurotoxic effects of AgNPs, many parameters, including particle size, shape, surface chemistry, protein corona profile, exposure route, dose, and duration, vary substantially. Such variability challenges the development of a consistent neurotoxicity profile for AgNPs. In particular, more comprehensive studies are needed to elucidate the neurological consequences of corona-dependent processes on the AgNP–neural tissue interaction. Moreover, most studies focus on short- or medium-term, relatively high-dose exposures; chronic low-dose exposures, environmental and occupational exposure scenarios, the developing or aging brain, and potential sex-related differences are addressed in a more limited manner. Thus, reliably optimizing experimental parameters becomes more difficult, and the development of a robust, neurotoxicity-focused risk assessment framework for AgNPs is delayed.

Despite these neurotoxic risks and limitations, AgNPs should not be considered only as potential risk agents. When appropriately designed, they also emerge as agents that may be considered for neurological therapeutic applications. Their ability to cross or affect the BBB under certain conditions, adjustable surface features, and strong antimicrobial effects make AgNPs potential candidates for drug delivery systems targeting the CNS, neuroimaging applications, biosensors, and antimicrobial coatings for neural implants. However, future AgNP-based systems should be developed by integrating the Safe-and-Sustainable-by-Design approach, rather than relying on toxicity evaluations conducted after material synthesis. This approach requires the simultaneous consideration of safety, functionality, environmental performance, exposure control, and practical feasibility. Particle size, shape, zeta potential, surface functionalization, colloidal stability, dissolution behavior, and Ag^+^ release should be defined as tunable design parameters. Modulating these variables may enable hazard reduction without compromising the targeted biological functionality. Furthermore, this approach should encompass the entire life cycle of AgNP-enabled products including the synthesis, manufacturing, use and end-of-life stages. Therefore, future studies should employ comparable assessment criteria that integrate physicochemical characterization with indicators of efficacy, human safety, environmental safety and sustainability. This design-driven strategy will facilitate the assessment of AgNP effects across complex biological systems under more controlled conditions and the interpretation of existing experimental findings in a broader biomedical context.

In conclusion, this review demonstrates that AgNPs have significant neurotoxic potential and should be thoroughly investigated to standardize their safer use. Elucidating the mechanisms underlying the observed toxicity and key parameters influencing it is essential. Although most current research centers on direct cellular interactions, the potential involvement of indirect systemic pathways, such as the gut–microbiota–brain axis, is emerging as a critical direction. A comprehensive understanding of both these direct and microbiota-mediated indirect mechanisms is crucial for predicting potential neurological complications in patients receiving future AgNP-based therapies. However, identifying preclinical risks alone is insufficient; these experimental findings must be translated into clinical protocols that safeguard patient safety. In this context, the findings synthesized in this review carry significant clinical implications. Evidence obtained from rodent models demonstrates that AgNPs are capable of inducing pronounced neurological effects within the CNS. Despite this, closing the knowledge gap arising from interspecies differences is a clinical necessity to ensure neurological safety in humans. Successfully addressing these translational barriers in future studies will be a decisive factor in determining the clinical applicability of AgNP-based neurotherapies. In this way, nanomedical innovations can be translated into clinical practice much more safely.

## Figures and Tables

**Table 1:** Summary of post-2020 in vitro and in vivo studies on AgNP-induced neurotoxicity.

Study Model	AgNP Properties	Exposure Condition and Route	Main Result	Link
In vitro (hESC-H9)	-Citrate-coated/stabilized AgNP (AgSC) -Size of 20 nm	-Exposure Route: added to culture media -Exposure Dose: 0.1 and 1.0 µg/mL -Exposure Rate: continuous exposure -Exposure Time: throughout neuronal initiation; until day 23	Disrupted neurogenesis; impaired rosette formation and reduced neural progenitor proliferation, with an altered neuron–astrocyte balance. Altered neurodevelopmental maturation; increased astrocyte activation with morphological changes and reduced neurite outgrowth and axon extension. Transcriptome-wide perturbation; disrupted metal homeostasis and cholesterol-related biosynthesis, with enrichment of neurodevelopmental and axon-guidance signaling pathways. Apoptosis- and stress-response pathway activation; enrichment of apoptosis-related processes and redox/stress programs. Ascorbic acid partially attenuated some adverse effects, whereas AgNO_3_ (1 ng/mL) did not reproduce astrocyte activation or neurite outgrowth effects	[100]
In vitro (SH-SY5Y; RA-induced neuronal differentiation model)	-Citrate-ligand-capped AgNP (nAg10) -Spherical shape with smooth surface -Size of 10 nm	-Exposure Route: added to culture media (co-treated with RA) -Exposure Dose: RA 10 μM; nAg10 0.02, 0.1, 0.5, 2.5 μg/mL -Exposure Rate: co-treatment; continuous exposure during incubation -Exposure Time: 72 h (primary endpoints) and 6 days (neurite morphology)	Suppressed RA-induced neuronal differentiation; reduced BDNF induction and neurite outgrowth in a dose-dependent manner. Impaired dopaminergic differentiation; reduced extracellular dopamine with a trend toward decreased TH/DAT expression. ROS-associated effect profile; increased cellular ROS with NAC-mediated restoration of BDNF expression Mitochondrial involvement; increased mitochondrial ROS and reduced mitochondrial fusion program, consistent with disrupted mitochondrial dynamics during differentiation.	[101]
In vitro (HMC3; SH-SY5Y co-culture)	-PVP-coated AgNP -Size of 5 nm	-Exposure Route: added to culture media (mono-culture); added to apical chamber (transwell co-culture) -Exposure Dose: 1 ng/mL–100 µg/mL (mono-culture dose–response); 1 µg/mL (transwell/mechanistic set) -Exposure Rate: continuous exposure during incubation -Exposure Time: mono-culture 24/48/72 h; transwell 24 h and 72 h; selected short exposures 90 min and 3/6/24/48 h.	Time- and dose-dependent cytotoxicity with oxidative stress; reduced cell viability with increased intracellular ROS in both HMC3 microglia and differentiated SH-SY5Y neurons. Inflammatory activation with DAM-like polarization; Toll-like receptor-4/NF-κB–linked pro-inflammatory signaling accompanied by a DAM-like activation profile, most evident after prolonged exposure. Nitric oxide dysregulation; time-dependent increase in NO production in HMC3 microglia Enhanced microglial phagocytic activity after prolonged exposure. Neuronal synaptic disruption; reduced SYN1 and SNAP-25 protein levels under co-culture conditions.	[102]
In vitro (hiPSC-derived cerebral organoids)	-PVP-coated AgNP -Spherical shape -Size of 20 nm	-Exposure Route: added to organoid maturation medium (6-well ultra-low attachment) -Exposure Dose: 0.1 and 0.5 µg/mL -Exposure Rate: continuous exposure during incubation -Exposure Time: 7 days (day 70–77); medium change every 2 days	Concentration-dependent developmental neurotoxicity; organoid size unchanged. Opposite effects on cell proliferation and apoptosis across concentrations Impaired ciliogenesis; disrupted cilium assembly/elongation, and ciliary motility at lower exposure. High-dose differentiation deficit; reduced neuronal/astrocytic differentiation features in VZ-like regions, with SOX2 largely unchanged. High-dose cytoskeletal disruption; altered actin/microtubule organization consistent with impaired neurite/growth-cone motility, with cytoskeleton-related transcriptomic enrichment.	[103]
In vitro (HT-22; mouse hippocampus neuron cell line) In vivo (BALB/c mice)	-PVP-coated AgNP -Spherical shape -Size of 5 nm (primary diameter) -Particle size of about 5–20 nm (dispersed in DMEM) -Zeta potential of −20.3 to −24.5 mV (in water)	-Exposure route: Addition to culture medium (DMEM + 10% FBS) in HT-22 cells and intranasal administration in BALB/c mice -Exposure dose: 20, 40, and 60 μg/mL in HT-22 cells and 4 mg/kg in BALB/c mice -Exposure time: 24 h in HT-22 cells -Exposure rate: Twice weekly in BALB/c mice -Exposure duration: 12 weeks in BALB/c mice	Altered amyloidogenic APP processing; decreased cell viability/proliferation in HT-22 mouse hippocampal neuronal cells. Cellular AgNP uptake and ferroptosis-associated mitochondrial morphology; increased intracellular iron accumulation, disrupted iron transport system, and dose-dependent ferritinophagy with altered autophagic flux. Dose-dependent redox imbalance with impaired antioxidant defense; enhanced lipid peroxidation. Impaired learning and spatial memory in the MWM; histopathological injury in hippocampus and cortex in BALB/c mice. Activated amyloidogenic processing with increased Aβ levels; reduced synaptic plasticity in hippocampus and cortex. Disrupted brain iron homeostasis with iron accumulation; increased lipid peroxidation and reduced antioxidant defense.	[104]
In vitro (BV2; mouse microglia cell line) In vivo (ICR mice)	-PVP-coated AgNP -Spherical shape -Size of 23.44 ± 4.92 nm	-Exposure route: Addition to culture medium (DMEM + 10% FBS) in BV2 cells and intravenous injection in male ICR mice -Exposure dose: 5, 25, and 50 μg/mL in BV2 cells and 12 and 120 mg/kg in ICR mice -Exposure time: 24 h in BV2 cells -Exposure rate: Injections on days 1, 4, 7, and 10 in ICR mice -Exposure duration: Brain tissues collected 7 days after the last injection in ICR mice	Increased membrane damage and cytotoxicity; dose-dependent ROS overproduction and increased granularity in BV2 mouse microglia cells. Shift toward a pro-inflammatory phenotype; dose-dependent IL-1β secretion and NF-κB upregulation, and NF-κB nuclear translocation. Autophagy dysregulation; increased autophagy-related signals and altered autophagic flux with autophagosome/autolysosome accumulation. Lysosomal membrane permeabilization and lysosomal structural/functional damage. Increased neuroinflammation-related signals and microglial activation toward an M1-like phenotype in ICR mice brain tissue; no serious pathological abnormalities in cortex. Increased autophagy-related signals in brain tissue; autophagosome accumulation in the hippocampus at the higher AgNP dose.	[105]
In vitro (Human umbilical vein endothelial cells, HUVECs) In vivo (Zebrafish embryos/larvae; WT, Tg(flk:eGFP), Tg(Hb9:eGFP))	-AgNP powder (CAS No. 7440–22-4) -Size combination of 10–100 nm	-Exposure route: Addition to fresh culture medium containing AgNP in HUVECs; treatment in E3 embryonic medium containing AgNP in zebrafish embryos/larvae -Exposure dose: 1, 2, and 4 mg/L in both model -Exposure time: 72 h in HUVECs (cells seeded and cultured for 12 h before exposure) -Exposure duration: 24–96 h in zebrafish embryos/larvae (endpoint-dependent), starting at 4 hpf -Exposure solution renewal: refreshed every 24 h for zebrafish treatment	Suppressed endothelial cell migration and tube formation capacity in HUVECs at the highest AgNP dose. Dose-dependent Ag uptake and accumulation in zebrafish embryos after AgNP exposure. Induced neurodevelopmental abnormalities, including a dose-dependent small-eye phenotype and disrupted motor neuron/axon development. Locomotor performance declined at the highest AgNP dose. Vascular development declined with angiogenesis malformations and abnormal vascular patterning, most evident at the highest AgNP dose. Altered transcriptomic profiles with enrichment of neuroactive ligands–receptor interaction and Vegf signaling pathways.	[106]
In vivo (Wistar albino rats)	-AgNPs powder (CAS-No. 7440-22-4); PVP-dispersed AgNP -Spherical or semi-spherical shape -Size of 45–120 nm; average 83 ± 37 nm (TEM) -Zeta potential of −33 mV	-Exposure route: oral gavage -Exposure dose: 10 mg/kg/day and 30 mg/kg/day -Exposure duration: 28 days -Exposure condition: dissolved in saline solution; gavage volume 10 mL/kg	Dose-dependent AgNP accumulation in cerebellar cortex; cerebellar cortical disorganization with prominent vacuolation and focal neuronal loss. Granular-layer neuronal injury; darkly stained/heterochromatic nuclei and vacuolar changes in cerebellar islands/neuropil. Neuroglial and ultrastructural injury; swollen Bergmann astrocytes, mitochondrial damage, and rER dilation in cerebellar cells. Myelin sheath disruption/splitting in cerebellar nerve fibers; BBB-related ultrastructural disruption with widened perivascular spaces and basement-membrane separation. Dose-dependent activation of apoptosis-, oxidative stress– and inflammation-related responses; increased systemic inflammatory cytokine levels (predominantly at high dose).	[107]
In vivo (Wistar rats)	-*Cornus mas* L. extract–biosynthesized AgNP (AgNP-CM); biomolecule-capped AgNP -Spherical shape -Size range of 5–30 nm; highest percentage size distribution at 20 nm -Intense SPR band at 414 nm -Zeta potential of −31 mV	-Exposure route: oral gavage -Exposure dose: 200 µg/day (0.8 mg/kg body weight) and 375 µg/day (1.5 mg/kg body weight) -Exposure rate: daily administration -Exposure condition: administered in 0.5 mL saline solution -Exposure duration: 45 days; assessments at end of exposure (T45) and 15 days post-exposure (T60)	Dose- and time-dependent increase in anxiety-like behavior; more pronounced post-exposure Neuronal ultrastructural degeneration in frontal cortex and hippocampus; nuclear alterations with ER/mitochondrial injury and autophagy-lysosome changes. BBB-associated ultrastructural disruption; irregular capillaries/endothelial alterations and astrocyte end-foot vacuolization/lysis. Dose- and time-dependent cerebral cortical histopathology; dark/apoptotic neurons with pericellular edema and myelin vacuolization. Astrogliosis with altered astrocyte morphology; increased GFAP immunoreactivity and shortened processes (post-exposure). Region- and time-dependent oxidative stress and inflammatory alterations; increased lipid peroxidation, reduced catalase activity, disturbed glutathione balance, and cytokine-level changes.	[108]
In vivo (CD-1 rats)	-Citrate-coated AgNP -Spherical shape with smooth and homogeneous surfaces -Average size of 26.9 nm -UV-Vis maximum absorbance wavelength (λmax) at 422 nm	-Exposure route: oral gavage -Exposure dose: 100 mg/kg bw and 1000 mg/kg bw -Exposure duration: 28 days	Dose-dependent silver accumulation in brain tissue; decreased total antioxidant capacity. Altered brain biophysical and neurochemical measures; decreased electrical conductivity/relative permittivity and reduced AChE activity, dopamine, and serotonin. Cerebral cortical injury; pyknotic neurons with gliosis and neuropil vacuolation/spongiosis (focal hemorrhage at the high dose). Marked myelinated axon degeneration; myelin reduction with splitting/separation and loss of lamellar organization. Ultrastructural neurodegeneration; rER/mitochondrial injury with nanosilver deposits and synaptic disruption.	[109]
In vivo (C57BL/6 mice)	-Citrate-coated AgNP (sodium citrate–stabilized) -Highly dispersed and uniformly distributed spherical shape -Size of 18 ± 1.8 nm -Zeta potential of −32 ± 1.2 mV -SPR peak at 400 nm	-Exposure route: oral gavage to dams (maternal exposure) -Exposure dose: 3 mg/kg bw -Exposure rate: daily administration -Exposure condition: 200 µL (0.4 mg/mL); AgNP suspension adjusted to pH 6.5 -Exposure duration: 2 weeks prior to breeding and throughout gestation; ended at parturition	Long-term gut microbiota dysbiosis in offspring; treatment-related separation in β-diversity/community structure and persistent taxa shifts, with no clear change in α-diversity. Altered repetitive/anxiety-like neurobehaviors; increased head-dipping, reduced rearing duration, and increased immobility tendency. Mixed/inconclusive spatial navigation/learning outcomes; trial-day-dependent effects. Reduced resident microglial cell counts in the hippocampus; no clear change in neuronal counts. Increased body fat content with a slight increase in total water content.	[110]
In vivo (Zebrafish embryos/larvae; AB strain; Tg(elavl3:EGFP), Tg(kdrl:mCherry), Tg(coro1a:EGFP))	-PVP-coated AgNP -Spherical shape -Size of 5.18 nm -Zeta potential of −17.7 ± 1.8 mV -UV–vis peak absorption at 410 nm	-Exposure route: waterborne exposure -Exposure dose: AgNPs 10 µg/L; PSNPs 200 µg/L; PSMPs 200 µg/L (single exposures); AgNPs 10 µg/L + PSNPs 200 µg/L; AgNPs 10 µg/L + PSMPs 200 µg/L (co-exposures) -Exposure duration: 6 hpf–120 hpf (endpoint-dependent) -Exposure Solution Renewal: refreshed every 24 h	Coexposure potentiated AgNP-induced neurotoxicity (AN > AM; endpoint-dependent). Developmental and physiological impairment (yolk abnormalities, reduced heart rate, shortened body length), exacerbated under coexposure. Behavioral impairment; reduced spontaneous locomotion, altered light–dark activity patterns, and impaired acoustic/tactile responses, more evident under coexposure. Neurodevelopmental and neurovascular injury; disrupted neuronal development and intracranial/ocular vascular patterning, with AChE inhibition Oxidative stress with antioxidant depletion; neuroimmune activation and apoptosis-related responses.	[111]
In vivo (adult zebrafish; wild-type AB strain)	-PVP-coated AgNP -Spherical shape -Size of ~5 nm -Zeta potential of −11.4 mV -UV-Vis absorption peak at 403 nm	-Exposure route: trophic dietary exposure via feeding with AgNP-pre-exposed brine shrimp -Exposure dose: brine shrimp pre-exposed to 0.1 and 100 μg/L AgNPs -Exposure duration: 21 days (from 90 to 111 days post-fertilization, dpf) -Exposure rate: fed twice daily -Exposure condition: feeding amount 2.5% of body weight	Trophic transfer with silver bioaccumulation and biomagnification in zebrafish brain and liver. Organ-level toxicity; decreased brain weight with neuronal injury/loss, and increased liver weight with hepatocyte disorganization and necrotic lesions. Neurobehavioral disruption; reduced swimming performance, increased anxiety-like behavior, altered learning/memory, and reduced social/aggressive behavior. Neurochemical and stress-axis disturbance; increased neuronal apoptosis with altered dopamine/serotonin levels and HPA-axis dysregulation. Hepatic metabolic–redox–inflammatory imbalance with liver–brain axis disruption; altered lipid/redox status and inflammatory signals, increased BBB permeability, and enhanced brain Aβ load/levels.	[112]

## Data Availability

No new data were created or analyzed in this study.
